# Modelling patterns of pollinator species richness and diversity using satellite image texture

**DOI:** 10.1371/journal.pone.0185591

**Published:** 2017-10-03

**Authors:** Sylvia Hofmann, Jeroen Everaars, Oliver Schweiger, Mark Frenzel, Lutz Bannehr, Anna F. Cord

**Affiliations:** 1 Department of Conservation Biology, Helmholtz Centre for Environmental Research, Leipzig, Germany; 2 German Centre for Integrative Biodiversity Research Halle-Jena-Leipzig, Leipzig, Germany; 3 Department of Community Ecology, Helmholtz Centre for Environmental Research, Halle Sa., Germany; 4 Department of Architecture, Institute for Geoinformation and Surveying, Facility Management and Geoinformation, Dessau, Germany; 5 Department of Computational Landscape Ecology, Helmholtz Centre for Environmental Research, Leipzig, Germany; Universita degli Studi di Trento, ITALY

## Abstract

Assessing species richness and diversity on the basis of standardised field sampling effort represents a cost- and time-consuming method. Satellite remote sensing (RS) can help overcome these limitations because it facilitates the collection of larger amounts of spatial data using cost-effective techniques. RS information is hence increasingly analysed to model biodiversity across space and time. Here, we focus on image texture measures as a proxy for spatial habitat heterogeneity, which has been recognized as an important determinant of species distributions and diversity. Using bee monitoring data of four years (2010–2013) from six 4 × 4 km field sites across Central Germany and a multimodel inference approach we test the ability of texture features derived from Landsat-TM imagery to model local pollinator biodiversity. Textures were shown to reflect patterns of bee diversity and species richness to some extent, with the first-order entropy texture and terrain roughness being the most relevant indicators. However, the texture measurements accounted for only 3–5% of up to 60% of the variability that was explained by our final models, although the results are largely consistent across different species groups (bumble bees, solitary bees). While our findings provide indications in support of the applicability of satellite imagery textures for modeling patterns of bee biodiversity, they are inconsistent with the high predictive power of texture metrics reported in previous studies for avian biodiversity. We assume that our texture data captured mainly heterogeneity resulting from landscape configuration, which might be functionally less important for wild bees than compositional diversity of plant communities. Our study also highlights the substantial variability among taxa in the applicability of texture metrics for modelling biodiversity.

## Introduction

Assessing biodiversity is essential to the effective monitoring of ecosystems and central to the development of sustainable management strategies and conservation plans for both natural and cultivated areas at various spatial scales [1]. However, measuring species richness and diversity on the basis of a standardised field sampling effort represents a cost- and time-consuming method, in particular over broad spatial scales [2, 3]. Therefore, complementary approaches are required to minimize monitoring resources and to maximize *a priori* knowledge about biodiversity patterns and quality of an area [4].

Spatial heterogeneity of habitats, i.e. the variation of the area in spatial scale, has been recognized as an important determinant of species distributions and diversity as regions with higher heterogeneity typically provide greater numbers of ecological niches and therefore can potentially host more co-existing species (e.g. [5, 6]). Spatial heterogeneity can be estimated from available satellite remote sensing (RS) data, e.g. through relationships with categorical land-cover information [7] or through indicators of spectral variability (SV) following the spectral variation hypothesis [2]. The latter hypothesis states that spectral heterogeneity of a RS image correlates well with landscape structure and complexity, which are directly related to spatial habitat heterogeneity. Hence, RS information is increasingly used to model and understand species distributions in space and time and to predict biodiversity-rich sites (see [4, 8, 9] and references therein). However, the commonly used land-cover classification data ignore subtle variations within a given class and gradients between classes, i.e. variation in vegetation structure [10, 11], which, in turn, influences the distribution of biodiversity [5]. Moreover, land-cover information is typically limited in both spatial and temporal grain, and, therefore, may not be the most pertinent for different species. The use of spatial heterogeneity in the spectral signal can overcome some of the limits of land-cover classifications (e.g. thematic constraints), and can therefore be a suitable proxy for species diversity estimates (reviewed in [3]). Yet, there are other challenges associated with this approach. For example, the strength of relationships between SV and biodiversity can vary considerably with spatial scales, diversity indices used, locations, and the imagery type [12, 13].

Similar to spectral radiance information, RS-based texture measures provide spatially continuous and temporally consistent observations of the land surface and render within-class vegetation structure. The use of these metrics has been recognized as an important method for quantifying spatial heterogeneity in terms of the spatial distribution [14, 15]. Image texture, considered as a function of the spatial variation in pixel brightness, is commonly measured as first-order (occurrence) and second-order (co-occurrence) statistics [16]. The first-order measures are summary computations, such as mean and standard deviation. They describe the frequency distribution of pixel values in a defined neighbourhood (commonly implemented as kernel or a moving window), while second-order statistics are based on the probability of joint occurrence of two pixel-intensity values which are in certain inter-pixel distance and orientation. Many of these texture metrics have been successfully explored to assess biodiversity. However, studies are biased towards certain species group (mainly vascular plants and birds; [11, 17–19]). For insect communities, the applicability of satellite-derived texture metrics for modelling patterns of local biodiversity is poorly understood. However, other remote-sensing techniques, e.g. airborne and terrestrial laser scanning, have been successfully, although barely, applied for modelling composition and diversity of spiders and beetles [20, 21].

Here, we focussed on wild bees, which are one of the most important groups of pollinators in most terrestrial ecosystems and economic crops [22, 23]. Bees are highly sensitive to floral resource abundance and diversity and probably also to the presence of nesting sites [24], and have frequently been shown to respond strongly to landscape heterogeneity and management [25–27]. For example, many wild bees nest or hibernate in semi-natural habitats and visit agricultural fields mainly for foraging. Consequently, pollinator species richness decrease with increasing distance from natural or semi-natural habitats, such as field margins, species-rich grasslands or forests edges [25]. Thus, bees are assumed to profit from complex landscapes within their foraging distance, making this group particular useful for our purposes.

The objective of our study was to examine the utility of RS texture information for indicating patterns of pollinator diversity across small spatial extents. We particularly aimed to explore the ability of texture features based on the Normalized Differenced Vegetation Index (NDVI) derived from 30-m resolution Landsat imagery to capture spatial habitat heterogeneity and model local pollinator species richness and diversity. While our results indicate some support to satellite-derived texture metrics as indicators for patterns of biodiversity, they underline the high level of variation among taxa in terms of the predictability of diversity measures by RS texture metrics.

## Material and methods

### Ethics statement

Bee data were collected in accordance with German law. The study was approved and annual sampling permissions were provided by the local nature conservation authorities (RL-0174-V; issued by Federal Agency for Environmental Protection Saxony-Anhalt).

### Study area and bee monitoring data

**Fig 1:**
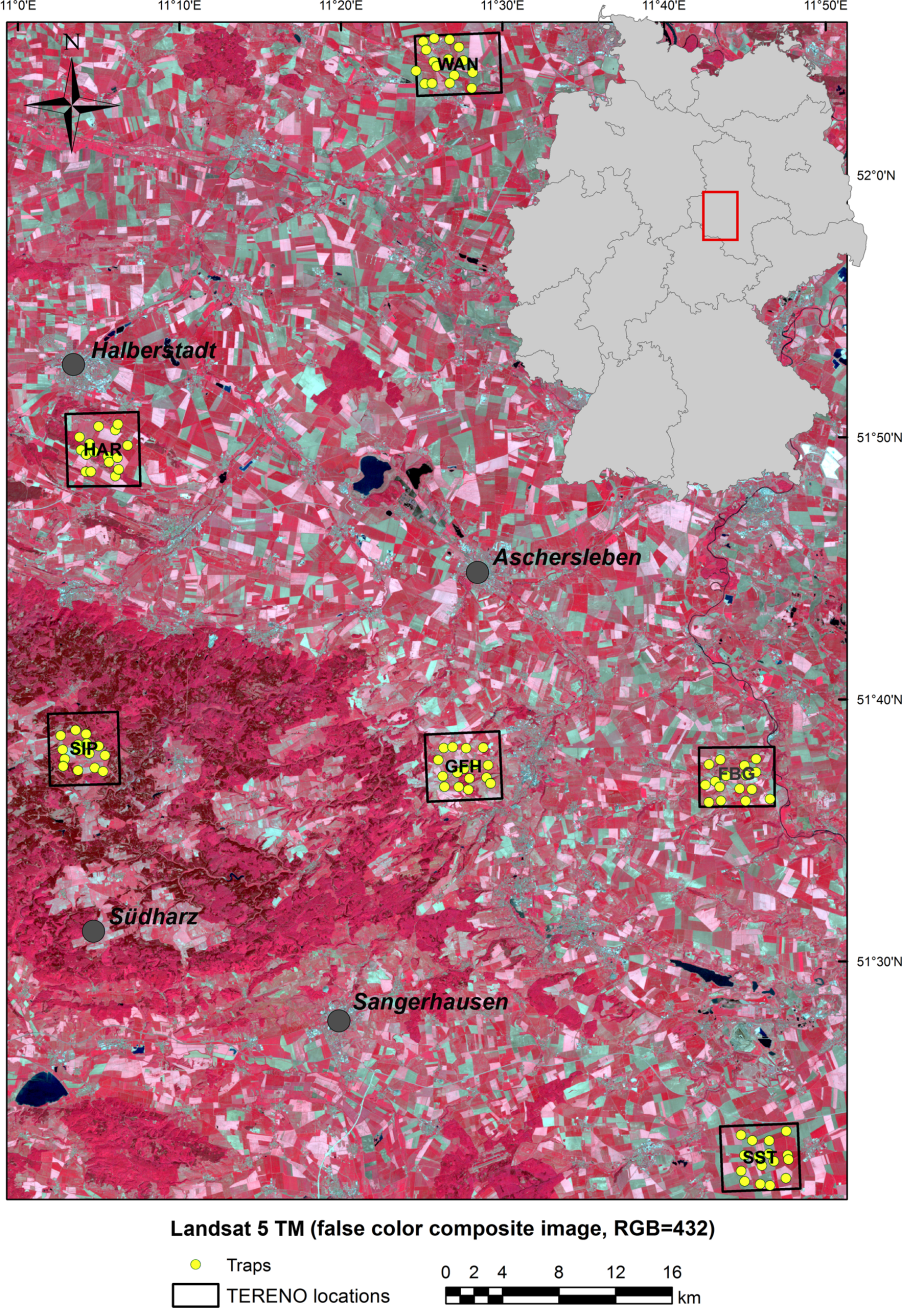
Landsat 5 TM image (Germany, Saxony-Anhalt, path 194, row 24, acquisition date: May 8^th^, 2011). The TERENO study locations are indicated by black frames (FBG: Friedeburg, GFH: Greifenhagen, HAR: Harsleben, SIP: Siptenfelde, SST: Schafstaedt, Wan: Wanzleben), and trapping points are given as yellow filled circles.

Bee monitoring data were used from six sites (Fig 1) across Saxony-Anhalt, Germany, generated in four consecutive years (2010–2013). The study sites are monitored on a regular basis as part of the TERENO project (Terrestrial Environmental Observatories; www.tereno.net) and of the German-European Long-Term Ecological Research network (LTER-D; http://www.ufz.de/lter-d/). They are representative for the dominating agricultural land use in a wider landscape and largely differ in terms of landscape structure and altitude (for details see Table 1). In particular, the region is characterized by a high variation in land-use intensity (from flat regions with up to 98% agriculture and large fields to regions with high levels of altitudinal heterogeneity, high cover of forests or other semi-natural habitats, less agriculture and smaller fields) and some variation in climatic conditions. The main crops are winter cereals, oilseed rape, maize and to some extent potato, sugar beet and peas [28]. Each site covers an area of 4 × 4 km and is divided in a grid of 1 × 1 km, containing one combined flight trap (a combination of yellow funnel and window panel; [29]) per grid cell. The traps were placed randomly within each square with the constraint that all traps had to be located at ecotones (i.e. transition zone between two habitat types, usually between an agricultural and a semi-natural area). Trapping efficiency was proofed extremely high and confirmed by local experts [30, 31]. Pollinator species were collected in two periods per year, extending from May to June (early summer) and from August to September (late summer), comprising 42.5 trapping days on average per period (41–50 days). Traps were active for two weeks before being emptied. Collected bees were identified to species level. Honeybees were excluded from the analyses to avoid potential anthropogenic effects caused by honeybee management. For an overview of the workflow of the study see S1 Fig.

**Table 1:** Coordinates (site centroids) and characteristics of the six study sites as specified in a previous work [28]. SN = semi-natural areas; CF = crop fields; For = forest; GL = grassland.

Site	Latitude	Longitude	Elevation (±SD)	SN	CF %	For %	GL %
**Friedeburg (FBG)**	51°6177°N	11°7096°E	122 (±31)	10	71	3	8
**Greifenhagen (GFH)**	51°6329°N	11°4340°E	270 (±27)	6	71	12	6
**Harsleben (HAR)**	51°8423°N	11°0753°E	143 (±14)	17	67	13	1
**Siptenfelde (SIP)**	51°6491°N	11°0526°E	423 (±31)	15	18	61	4
**Schafstaedt (SST)**	51°3770°N	11°7224°E	177 (±11)	2	97	0.3	0.1
**Wanzleben (WAN)**	52°0803°N	11°4518°E	113 (±10)	8	77	4	3

### Remote sensing data sources and image metrics processing

The red (band 3) and near-infrared (band 4) from a 30-m resolution Landsat-5 TM scene acquired on May 08, 2011 (path 194, row 24) were used to calculate the NDVI as implemented in ENVI 5.0 software (Research Systems Inc., Boulder, CO). Atmospheric correction of the Landsat data was performed using the Landsat Ecosystem Disturbance Adaptive Processing System (LEDAPS; [32]). The Landsat image was captured during a time that matches the early sampling period of the pollinators at the TERENO sites. Previously published research has been shown that NDVI texture is superior to the texture of any individual Landsat TM band for modelling biodiversity [15]. Based on this NDVI image a set of first- and second-order texture measures was computed in ENVI 5.0, except evenness (see below), including metrics that have been recently proven to be particular suitable to capture spatial habitat heterogeneity [33]: mean, evenness, entropy and variance (first-order textures) as well as contrast, dissimilarity, entropy and homogeneity (second-order textures); see Table 2. Evenness was calculated with the *r*.*diversity* module using GRASS GIS 7.2.0 [34, 35]. Second-order texture measures were extracted from a grey-level co-occurrence matrix (GLCM), a tabulation of how often different combinations of pixel brightness values occur in the image [16, 36]. Since this matrix is sensitive to rotation we varied the directions for texture calculations by four different rotational angels (0°, 45°, 90° and 135°), and averaged the results [16]. All image texture analyses were computed from a window of 3 × 3 pixels (90 m × 90 m), which was considered an adequate size for measuring neighbour conditions relevant to pollinators. In addition to the mean of the textures, we selected the measure of first-order standard deviation due to its 'intuitive appeal' for characterizing levels of heterogeneity [18]. In order to relate our measures to the different foraging distances of the various pollinator groups we derived different-sized ring buffers around each sample site (100–1000 m, 100 m intervals). For each buffer area we then calculated the mean and standard deviation of each of the metrics using Hawth’s tool [37], as well the mean and coefficient variation (cv) of the NDVI. Moreover, we derived a microtopography-based 'terrain roughness' metric from a local 10 m digital elevation model (DGM 10, GeoBasis-DE/BKG 2012), that used the same coordinate system as the other layers (ETRS89/UTM zone 32N). Roughness is a measure of spatial configuration or landscape heterogeneity, as it is correlated with other terrain attributes (e.g. relief, standard deviation of elevation, slope and standard deviation of slope). We generated this metric as the amount of elevation difference between adjacent cells according to [38] and [39] using the Geomorphometry and Gradient Metrics Toolbox v2.0 [40] and applying a moving window size of 9 × 9 (i.e. 90 × 90 m). Hawth’s tool was again used to summarize roughness values (mean, standard deviation) within the different buffer areas around each sample point.

**Table 2:** The metrics generated in the study as measures of spatial heterogeneity.

Metric	Measure	Formula [16, 41–45]
Mean of NDVI	Mean of the NDVI values within the buffer areas	$\bar{NDVI}$
Coefficient of variation	Normalized dispersion of NDVI within the buffer areas	$\frac{SD[NDVI]}{\bar{NDVI}}$
**1**^**st**^ **order textures**		
Mean	Mean value of NDVI of the processing window	$\sum_{i=0}^{Ng-1}i\;P(i)$
Entropy	Disorder of NDVI	$-\sum_{i=0}^{Ng-1}P(i)ln[P(i)]$
Evenness	Evenness of NDVI	$\frac{-\sum_{1=1}^{Ng}P(i)ln[P(i)]}{ln(Ng)}$
Variance	Dispersion of the NDVI values around the mean	$\sum_{i=0}^{Ng-1}{\left(i-M\right)}^{2}P(i)$
**2**^**nd**^ **order textures**		
Contrast	Exponentially weighted difference in NDVI between adjacent pixels	$\sum_{i=1}^{Ng}\sum_{j=1}^{Ng}P(i,j){\left(i-j\right)}^{2}$
Dissimilarity	Difference in NDVI between adjacent pixels	$\sum_{i=1}^{Ng}\sum_{j=1}^{Ng}P(i,j)|i-j|$
Entropy	Disorderliness of NDVI	$-\sum_{i}\sum_{j}P(i,j)log(P(i,j))$
Homogeneity	Uniformity of NDVI between adjacent pixels	$\sum_{i=1}^{Ng}\sum_{j=1}^{Ng}\frac{1}{1+{\left(i-j\right)}^{2}}P(i,j)$
Roughness	Elevation difference between adjacent cells of a DEM	$Y{\left[\sum (x_{ij}{-x_{00})}^{2}\right]}^{\frac{1}{2}}$

Ng = total number of distinct grey levels in the window; P(i) = proportion of occupancy of each pixel value; *x*_*ij*_ = elevation of each neighbour cell to cell (0,0); for ArcGIS source code of roughness see [39].

### Pollinator data: Rarefaction and diversity analyses

Data preparation and all analyses were performed in R version 3.2.5 [46]. Because we did not expect all pollinator species to respond uniformly to measures of textures, we performed all analyses on (i) the whole community of wild bees (dataset 'nohb') and the community split in two subsets; ii) bumble bees (data set 'bb') and iii) remaining wild bees (data set 'sb'). This roughly separates the community in large eusocial bees and smaller bees that are mostly solitary. Although the second subset is not a 'pure' trait group, this separation is often used to identify differences relating to foraging distance (body size) and food requirements (whole colony at once, or individual, solitary, foragers) [47, 48]. In addition to the number of bees (bee count, BC; normalized to trapping days), we estimated abundance-independent species richness (SpR) by rarefaction with the iNext package [49, 50]. Species richness was interpolated to three times the minimum abundance of individuals in the dataset. Shannon’s diversity (*SD*) [51] was estimated using the 'Hs' function in the DiversitySampler package [52].

Although BC, SD and SpR were partly positively correlated (S1 Table), we analysed them separately following the same procedure. The three data sets were initially explored through descriptive analysis. Pollinator data (BC, SD, SpR) was assessed per season and year in all three datasets (see above) for normal distribution using Shapiro-Wilk tests and Q-Q plot analyses. Annual, seasonal and location-associated differences were tested within each biodiversity measurement using the Kruskal-Wallis test [53]. Finally, we used Pearson’s correlation coefficients (r) of the season classes 'early' and 'late' to determine whether there was a strong relationship between them, which would provide additional evidence for a fixed seasonal effect.

### Statistical analysis

**Fig 2:**
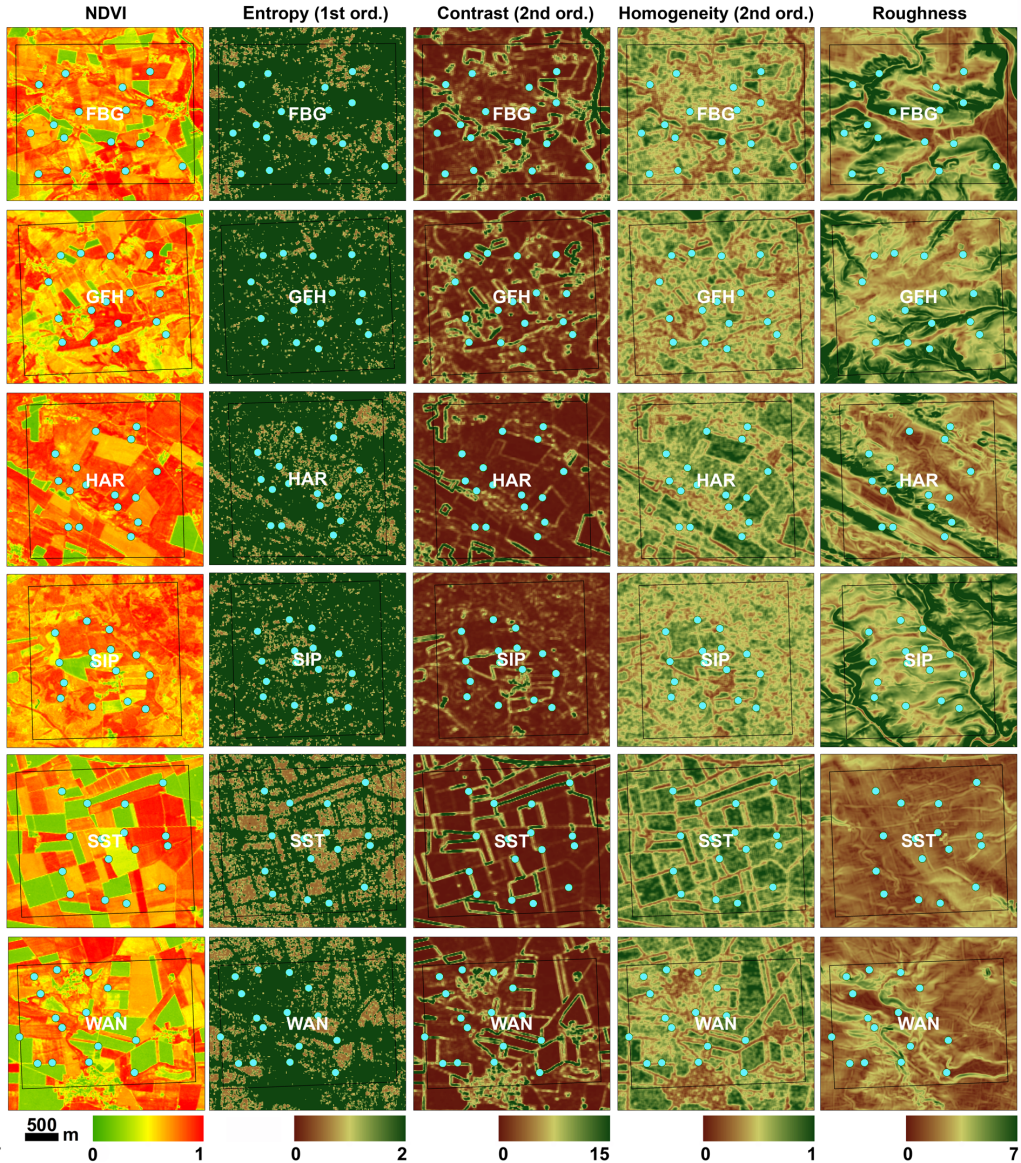
Spatial habitat heterogeneity within the six study sites (FBG, GFH, HAR, SIP, SST, WAN) captured by the presented texture metrics. Light blue dots correspond to the trapping points.

To eliminate predictor collinearity prior to generating the models, we first calculated Pearsons’s correlation coefficients for all pairs of distance classes per variable to remove classes with coefficients │c│> 0.7 (S2 Fig). We eliminated all data of correlated distance classes and all variables that showed a correlation across all distance classes. Secondly, we computed the correlation between the remaining variables and excluded the variable of a correlated pair with │c│> 0.7 that we considered to be the less biologically important of the two (S3A and S3B Fig). The resulting dataset contained two distance classes (100 m and 1000 m) each with five potentially predictive variables (cvNDVI, roughness, entropy [1^st^ order], contrast and homogeneity [2^nd^ order]; Fig 2). Further potential correlations between variables within distance classes were addressed during model building.

To investigate the potential relations of biodiversity to image texture indices, we applied linear models (LM). We used the R-packages car [54], MASS [55], MuMln [56], ncf [57] and nlme [58]. For each dataset (bb, sb, nohb) and response variable (BC, SD, SpR) we first built a full model by including all texture metrics as fixed effects test predictors as well as the location site, season and year as fixed effects control predictors. Control predictors were not pertinent to our hypotheses but have known effects that needed to be controlled for to allow valid conclusions about our test predictors [59]. We calculated the variance inflation factors (vif) for each of the full models and excluded predictors with a square root of the vif > 2 [60]. Due to their high vif the metrics homogeneity, entropy and roughness for the 1000 m distance class as well as contrast for the 100 m distance class were excluded from the full model (Table 3 for an overview of all predictors throughout the models, and S4 Fig). Model residuals were inspected to assess model fit. We ensured that model residuals were not spatially autocorrelated by creating spline correlograms [61] using the *ncf* package in R. The significance of correlograms was tested for distance classes increasing by 100 m using randomization test with 500 times of permutation at the 0.05 level.

**Table 3:** Overview of predictors used in the averaged LMs. 1^st^ = first order texture; 2^nd^ = second order texture; bb = bumble bees; cv = coefficient of variance; con = contrast; ent = entropy; hom = homogeneity; nohb = all wild bees; rough = roughness; sb = solitary bees; BC = bee count; SD = Shannon’s diversity; SpR = Species richness.

		Fixed effects test predictors						Fixed effects control predictors		
		1^st^ ent 100 m	2^nd^ hom 100 m	cv NDVI 100 m	rough 100 m	2^nd^ con 1000 m	cv NDVI 1000 m	location	year	season
**BC**	bb	x	x	x	x	x	x	x	x	x
	nohb	x	x	x	x	x	x	x	x	x
	sb	x	x	x	x		x	x	x	x
**SD**	bb	x	x	x	x	x	x	x	x	x
	nohb	x	x		x	x		x	x	x
	sb	x	x	x	x	x	x	x	x	x
**SpR**	bb	x	x	x	x		x	x	x	x
	nohb	x	x	x	x		x	x	x	x
	sb	x	x		x	x	x	x	x	x

To assess the impact of individual image metrics, we generated models with all possible combinations of predictors of the global model, as we did not have any *a priori* hypotheses on the subsets of predictors in question. We did not fit interactions between any of the factors, as this would require a large number of coefficients and would be difficult to interpret. All test predictors were scaled to allow comparing their relative effects. Models on the different biodiversity variables (BC, SD, SpR) were compared using Akaike’s information criterion (AIC; [59, 62]). Statistical differences between models were based on ΔAIC scores larger than 2 [59]. Parameter estimates and standard errors were obtained as model-averaged estimates from the top model set (ΔAIC ≤ 2), and their p‐values with likelihood ratio tests (LRT) of the full model against the model without the effect in question. Finally, we constructed the null model (including only the control predictors) and compared it to the global model to assess the amount of variance that is explained by the test and the control predictors, respectively.

## Results

### Pollinator data characteristics

During four years of monitoring, more than 20.000 individual bees of 260 bee species were collected. 238 out of these taxa were solitary bee and 22 bumble bee species. In most of the subsets, the data showed a significant deviation from a normal distribution for seasons, except for the log-transformed bee count and species richness of the full data and the solitary bees (data not shown). Highly significant differences in biodiversity were present between seasons and locations in the bumble bees, solitary bees and wild bees data set (S5 and S6 Figs). Again, bee count and species richness showed no differences between years in the full and solitary-bee data sets but in the bumble-bee data. Pearson’s correlation coefficients (r) of the season classes 'early' and 'late' ranged between 0.14 and 0.42 with p-values lower than 0.01, providing indications for a systematic seasonal effect on bee diversity (S7 Fig).

According to the spline correlograms the fitted residuals of the global LMs showed no evidence for significant spatial structure among the traps (S8 Fig). This was supported by the randomization tests that did not show any significant autocorrelation whatever the distance lag considered (data not shown). Therefore, we considered the residuals as spatially independent.

### Species richness and diversity models

For each of the three data sets (BC, SD, SpR), the LM explained at least 15% of the variability of biodiversity with an R^2^ ranging from 0.149 (species-richness model of solitary bees) to 0.596 (bumble bee count model; Table 4). Five of the image measures (roughness, cv of NDVI, 1^st^ order entropy and 2^nd^ order homogeneity within 100 m radius as well as cv of the NDVI within 1000 m radius), were included in seven of the nine optimal models selected by AIC. Although most of the variability was captured by the control predictors (location, year, season), the texture metric entropy (1^st^ order) and/or the roughness of the surface contributed significantly to each of the models (Table 4). Notably, surface roughness remained in every model used for model averaging, while entropy remained in every model except for the species richness of bumble bees (S2 Table). Regardless of the data set, our image metrics performed slightly better in the Shannon’s-diversity and species-richness models (ΔR2- R^2^NULL = 0.031 to 0.048) compared to the bee count models (ΔR^2^- R^2^NULL = 0.01 to 0.036). However, the overall model performance of the species-richness model in solitary bees (R^2^ = 0.206) and the full data set (0.149) was substantial lower than for the Shannon’s diversity (R^2^ = 0.46 and 0.49) and bee count models (0.52 and 0.60).

**Table 4:** Model-average estimates (EST) of scaled test predictors of bee biodiversity represented by bee count (BC), Shannon diversity (SD), and species richness (SpR) using bumble bees (bb), solitary bees (sb) and all wild bees (nohb). The R^2^ corresponds to the global model including all predictors that remained after model selection, while the R^2^ of the Null model (R^2^_NULL_) refers to the model including only the fixed effects control predictors. Δ = R^2^- R^2^_NULL_. IMP = relative importance; SD = Standard deviation; n_model_ = number of models averaged; P = P-values. con2 = 2^nd^ order contrast; ent1 = 1^st^ order entropy; hom2 = 2^nd^ order homogeneity; NDVI_cv = coefficient of variance of the NDVI; rough = surface roughness; 100/1000 = 100 m or 1000 m scale.

**(bb)**	**BC (log), n**_**model**_ **= 6**				**Shannon Diversity, n**_**model**_ **= 8**				**Species Richness, n**_**model**_ **= 6**			
**Test predictor**	EST	SD	P	IMP	EST	SD	P	IMP	EST	SD	P	IMP
**con2_1000**	-0.021	0.029	0.458	0.51	-0.028	0.038	0.453	0.51				
**ent1_100**	0.051	0.019	**0.006**	1.00	0.065	0.023	**0.005**	1.00	0.013	0.051	0.803	0.15
**hom2_100**	0.060	0.020	**0.003**	1.00	0.008	0.018	0.666	0.30	0.006	0.041	0.882	0.12
**NDVI_cv_100**	-0.005	0.014	0.740	0.23	-0.001	0.009	0.900	0.08	-0.037	0.091	0.685	0.28
**NDVI_cv_1000**	0.012	0.023	0.613	0.39	0.025	0.035	0.471	0.54	0.033	0.100	0.742	0.25
**roug_100**	0.073	0.021	**<0.001**	1.00	0.101	0.027	**<0.001**	1.00	0.513	0.125	**<0.001**	1.00
	R^2^ = 0.220; R^2^_NULL_ = 0.184; ^2^ = 0.036				R^2^ = 0.192; R^2^_NULL_ = 0.155; Δ = 0.037				R^2^ = 0.215; R^2^_NULL_ = 0.179; Δ = 0.036			
**(sb)**	**BC (log), n**_**model**_ **= 8**				**Shannon Diversity, n**_**model**_ **= 8**				**Species Richness, n**_**model**_ **= 6**			
**Test predictor**	EST.	SD	P	IMP	EST	SD	P	IMP	EST	SD	P	IMP
**con2_1000**					0.003	0.012	0.814	0.18	0.120	0.150	0.427	0.53
**ent1_100**	0.054	0.018	**0.002**	1.00	0.095	0.022	**<0.001**	1.00	0.380	0.091	**<0.001**	1.00
**hom2_100**	0.005	0.013	0.725	0.23	0.017	0.024	0.486	0.49	0.045	0.083	0.587	0.39
**NDVI_cv_100**	-0.018	0.021	0.390	0.61	-0.011	0.020	0.584	0.37				
**NDVI_cv_1000**	0.002	0.012	0.859	0.15	-0.003	0.015	0.839	0.16	-0.126	0.144	0.383	0.59
**roug_100**	0.024	0.024	0.322	0.67	0.087	0.025	**<0.001**	1.00	0.227	0.101	**0.025**	1.00
	R^2^ = 0.596; R^2^_NULL_ = 0.587; Δ = 0.010				R^2^ = 0.493; R^2^_NULL_ = 0.464; Δ = 0.029				R^2^ = 0.206; R^2^_NULL_ = 0.165; Δ = 0.041			
**(nohb)**	**BC (log), n**_**model**_ **= 5**				**Shannon Diversity, n**_**model**_ **= 3**				**Species Richness, n**_**model**_ **= 4**			
**Test predictor**	EST.	SD	P	IMP	EST	SD	P	IMP	EST	SD	P	IMP
**con2_1000**	-0.001	0.009	0.877	0.14	0.010	0.020	0.662	0.30				
**ent1_100**	0.045	0.016	**0.005**	1.00	0.112	0.021	**<0.001**	1.00	0.300	0.069	**<0.001**	1.00
**hom2_100**	0.008	0.016	0.599	0.36	0.033	0.025	0.196	0.79	0.061	0.072	0.396	0.20
**NDVI_cv_100**	-0.028	0.020	0.160	0.83					-0.041	0.069	0.551	0.17
**NDVI_cv_1000**	0.002	0.010	0.848	0.15					-0.081	0.085	0.340	0.23
**roug_100**	0.042	0.019	**0.023**	1.00	0.104	0.022	**<0.001**	1.00	0.218	0.081	**0.007**	1.00
	R^2^ = 0.525; R^2^_NULL_ = 0.509; Δ = 0.016				R^2^ = 0.458; R^2^_NULL_ = 0.410; Δ = 0.048				R^2^ = 0.149; R^2^_NULL_ = 0.118; Δ = 0.031			

## Discussion

Our models revealed that texture metrics derived from Landsat imagery played a significant but minor role in explaining local pollinator biodiversity, with the first-order entropy texture and terrain roughness being the most relevant indicators. The results are in striking contrast to the high predictive power of texture metrics reported previously for avian communities.

### Pollinator biodiversity, texture metrics and habitat heterogeneity

The results of the present study indicate some support for existing evidence that texture metrics represent a potential tool for modelling biodiversity. We found that on small spatial scale (within 100 m) first order entropy and surface roughness explained a substantial proportion (up to 5%) of the variability in pollinator count, species richness and Shannon’s diversity, with our final models explaining between 15% and 60% of the variability. Our findings are largely consistent with each other, regardless of the defined taxa group, suggesting that the methods we used and the results we present are robust. Similar low but likewise significant levels (4%) of explained variance by texture information were recently reported for biodiversity models of Arctiinae moths [63].

In previous studies, various texture features have been proposed as best predictor for biodiversity at different spatial scales and for different habitat types, such as mean summary of NDVI values for ovenbird density and occurrence [64], the angular second moment for species richness and diversity of shrub and tree nesting bird species [18], and second order homogeneity for avian species richness in diverse habitat types [33]. Entropy, however, hasn’t attracted much attention so far, although in most of the studies using texture metrics for the analyses of biodiversity patterns, first-order entropy is among the set of 'standard' measurements [11]. In our study, this metric accounted for a significant amount of variance in all biodiversity indices within the different datasets, except for species richness of bumble bees. Species richness typically does not consider species abundances, but rather the number of species in a particular area. As the number of bumble bee species present on our sites was considerably lower than in solitary bees, their variance might have been too small to be captured to some degree by texture metrics.

Entropy in any system represents uncertainty, where in the case of texture analysis it characterizes spatial ‘disorderliness’ of an image [16] and is inversely related to measures of local homogeneity (e.g. the angular second moment or energy). Our study sites are dominated by agricultural land use, but differ largely in terms of altitude and landscape structure (e.g. 2–17% cover of semi-natural areas; Table 1). Farmland fields usually occur as homogeneous patches of similar grey values in the image, while any discontinuity would increase heterogeneity among grey values, leading *a priori* to a higher number of distinct tone levels within the moving window during analyses, and, thus, higher entropy. Ultimately, the metric relates significantly to our underlying hypothesis that biodiversity can be modelled by local heterogeneity. Our results line up with previous findings showing that entropy is among the most sensitive texture metric to map farmland field size and as such the degree of local heterogeneity [65]. Consistently, entropy statistics of the NDVI have been shown to depict the diversity of vegetation types, which is assumed to be directly related to structural complexity, thus, may support more coexisting species [63]. Since wild bees’ abundance and species richness have been shown to be enhanced by landscape heterogeneity (percentage of semi-natural habitats) [66], it is not surprising that the entropy texture measurement was able to explain some of the variance in our models.

Similar to first-order entropy, surface roughness accounted for a significant proportion of the variance of bee count, diversity and richness (with the only exception being the abundance of solitary bees). The linkage between topographic characteristics and species distribution across different spatial scales is a well-known fact ([67, 68] and references therein) that has been used for modelling and monitoring biodiversity, especially in plants (e.g. [68–71]), but also in birds [28, 72]. Moreover, relief roughness has been demonstrated to be strongly related to land-use intensity. The latter decreases rapidly with an increasing variability of the surface area, which is accompanied by increasing landscape heterogeneity that, in turn, impacts biodiversity [73, 74]. Again, pollinator communities have been shown to benefit from heterogeneous landscapes because of both their structural as well as plant mediated positive effects on bee diversity [26]. For example, nesting sites are almost exclusively found in (semi-)natural habitats, underlining the importance of a heterogeneous agri-environment [75]. We assume that plain and smooth surfaces are characteristic for larger homogeneous agricultural segments that are intensively managed, providing less complex environments and therefore harbour less diverse communities where generalist species dominate. In contrast, landscapes characterized by a hilly surface are likely to have smaller agricultural patches and a mixture of different land cover types, associated with lower land use intensity and higher natural heterogeneity [74].

### Relevance of the results and limitations of the study

Although the consistency of our results in the different data sets provides indications in support of the potential of the two metrics (entropy, roughness) for modelling pollinator diversity and richness, the relatively low amount of variability explained by textures in our models differs considerably to previous findings in bird communities. Measures of image texture were highly effective in modelling spatial patterns of avian biodiversity in multiple habitat types, accounting for over 50% (7–51%) or even for up to 82% (31.5–82.3%) of the variability in species richness [15, 17]. These variations in the ability of texture metrics to predict biodiversity corroborate recent contributions, showing that the applicability of indicators developed from texture analysis differ significantly among taxa [63].

Bees respond very differently to landscape features compared to birds, and textures information probably depict only minor parts of features that bees are responding to. For example, (foraging) plant species composition and flower abundance [26, 76] might be functionally more important for wild bees than geometric complexity of spatial patterning of vegetation cover types [66]. In addition, wild bees are assumed to respond more strongly to specific habitat features at local scale than to landscape configuration at larger spatial scales [77]. However, the resolution of most sensors currently employed for RS derived texture analyses has so far prevented the examination of the internal heterogeneity of plant communities, as the size of commonly-used pixels is too large to detect such small-scale variation [78]. Hence, the grain of Landsat data may not be sufficient for echoing the (most) relevant habitat components of pollinator species (e.g. nesting sites, flower diversity). In contrast, many birds respond strongly to vegetation/habitat structure, while plant species composition is often less or equally important, particularly over large spatial scale (landscape level) [79–81]. Therefore, avian communities represent a biological group that seems to be far more pertinent compared to pollinators to be modelled by image textures in terms of species richness, diversity and abundance. In pollinators, image texture parameters alone appear to be less applicable for the predictability of diversity measures. Yet, they may improve predictions of species diversity in more sophisticated models [63].

Knowing whether image texture information are differently useful in different taxonomic groups, and which metrics are more useful than others in modelling biodiversity is important for a number of reasons. From a practical perspective these insights will help guiding researchers as to which metrics and which (meaningful) taxa they should focus on when assessing biodiversity by texture information. Also, this knowledge will add to a better understanding at which scale relevant biological properties, to which the respective species community is responding, can be depicted by textural attributes.

## Conclusion

Our results provide some support for previous findings that texture metrics derived from Landsat and DEM data can indicate patterns of species diversity and richness by reflecting spatial heterogeneity. However, the limited performance of the texture metrics in our models (in particular in comparison to similar studies in avian biodiversity) underlines both the substantial variation among taxa in terms of the applicability of these measures and an insufficient spatial resolution. Other, for pollinator species potentially more relevant information, such as compositional heterogeneity of ground vegetation, was possibly not reflected by textural attributes at the given resolution. It is hoped, that recently launched sensors (e.g. Sentinel-2) will provide habitat data with higher spatial and temporal resolution (compared to Landsat) that will probably greatly facilitate analyses as in the study at hand.

## Supporting information

S1 Fig Workflow of the study. (DOCX)

S2 Fig Pearsons’s correlation coefficients for all pairs of distance classes per test variable. (DOCX)

S3 Fig Pearsons’s correlation coefficients between the test variables (a) and remaining variables after removing variables with coefficients │c│≥ 0.7 (b).
(DOCX)

S4 Fig Pearsons’s correlation coefficients for all pairs of remaining test variable and distance class (100 and 1000 m). (DOCX)

S5 Fig Differences between trapping seasons (2010–23013) within the biodiversity variables per data set (df). (DOCX)

S6 Fig Differences between years (upper two rows) and locations (lower two rows) within the biodiversity variables per data set (df). (DOCX)

S7 Fig Pearson’s correlation of the early and late trapping season for the biodiversity variables per data frame (df). (DOCX)

S8 Fig Spline correlograms for the global fitted models as per model selection for each response variable of (a) the bumble-bees data set (bb), (b) the solitary-bees data set (sb), and (c) the wild bee data set (nohb).
(DOCX)

S1 Table Correlation between bee count (BC), Shannon’s diversity (SD) and species richness (SpR) variables. (DOCX)

S2 Table Model comparison for predictors of biodiversity in bumble bees (bb), solitary bees (sb), and all wild bees (nohb). (DOCX)

## References

[1] Henry PY, Lengyel S, Nowicki P, Julliard R, Clobert J, Celik T, et al Integrating ongoing biodiversity monitoring: potential benefits and methods. Biodivers Conserv. 2008;17(14):3357–82.

[2] Palmer MW, Earls PG, Hoagland BW, White PS, Wohlgemuth T. Quantitative tools for perfecting species lists. Environmetrics. 2002;13:121–37.

[3] Rocchini D, He K, Oldeland J, Wesuls D, Neteler M. Spectral variation versus species beta-diversity at different spatial scales: a test in african highland savannas. J Environ Monitoring. 2010;12:825–31. 10.1039/b921835a 20383362

[4] Rocchini D, Balkenhol N, Carter GA, Foody GM, Gillespie TW, He KS, et al Remotely sensed spectral heterogeneity as a proxy of species diversity: Recent advances and open challenges. Ecol Inform. 2010;5(5):318–29.

[5] MacArthur R, MacArthur J. On bird species diversity. Ecology. 1961;42(3):594–8.

[6] MacArthur RH, Wilson EO. The theory of island biogeography. Princeton: Princeton: University Press; 1967.

[7] Baldi G, Paruleo JM. Land-Use and land cover dynamics in South American temperate grasslands. Ecol Soc. 2008;13(2):6.

[8] Duro DC, Girard J, King DJ, Fahrig L, Mitchell S, Lindsay K, et al Predicting species diversity in agricultural environments using Landsat TM imagery. Remote Sens Environ. 2014;144:214–25.

[9] Rocchini D, Boyd DS, Féret J-B, Foody GM, He KS, Lausch A, et al Satellite remote sensing to monitor species diversity potential and pitfalls. Remote Sens Ecol Conserv. 2016 01; 2(1):25–36.

[10] St-Louis V, Pidgeon AM, Radeloff VC, Hawbaker TJ, Clayton MK. High-resolution image texture as a predictor of bird species richness. Remote Sens Environ. 2006;105:299–312.

[11] Wood EM, Pidgeon AM, Radeloff VC, Keuler NS. Image texture as a remotely sensed measure of vegetation structure. Remote Sens Environ. 2012;121:516–26.

[12] Rocchini D. Effects of spatial and spectral resolution in estimating ecosystem alpha-diversity by satellite imagery. Remote Sens Environ. 2007;111(4):423–34.

[13] Herkul K, Kotta J, Kutser T, Vahtmae E. Relating remotely sensed optical variability to marine benthic biodiversity. PLOS One. 2013;8(2): e55624 doi: 10.1371/journal.pone.0055624 23405180 10.1371/journal.pone.0055624 PMC3566085

[14] Culbert PD, Pidgeon AM, St-Louis V, Bash D, Radeloff VC. The impact of phenological variation on texture measures of remotely sensed imagery. Ieee J-Stars. 2009;2(4):299–309.

[15] St-Louis V, Pidgeon AM, Clayton MK, Locke BA, Bash D, Radeloff VC. Satellite image texture and a vegetation index predict avian biodiversity in the Chihuahuan Desert of New Mexico. Ecography. 2009;32:468–80.

[16] Haralick RM, Shanmuga K, Dinstein I. Textural features for image classification. IEEE Transactions on Systems, Man, and Cybernetics. 1973;SMC-3:610–21.

[17] Culbert PD, Radeloff VC, V S-L, Flather CH, Rittenhouse CD, Albright TP, et al Modeling broad-scale patterns of avian species richness across the Midwestern United States with measures of satellite image texture. Remote Sens Environ. 2012;118:140–50. doi: 10.1016/j.rse.2011.11.004

[18] St-Louis V, Pidgeon AM, Kuemmerle T, Sonnenschein R, Radeloff VC, Clayton MK, et al Modelling avian biodiversity using raw, unclassified satellite imagery. Philos Trans R Soc Lond B Biol Sci. 2014;369:1471–2970. 10.1098/rstb.2013.0197 PMC3983932 24733952

[19] Hernandez-Stefanoni JL, Gallardo-Cruz JA, Meave JA, Rocchini D, Bello-Pineda J, Lopez-Martinez JO. Modeling alpha- and beta-diversity in a tropical forest from remotely sensed and spatial data. Int J Appl Earth Obs. 2012;19:359–68.

[20] Muller J, Brandl R. Assessing biodiversity by remote sensing in mountainous terrain: the potential of LiDAR to predict forest beetle assemblages. J Appl Ecol. 2009;46(4):897–905.

[21] Vierling KT, Bassler C, Brandl R, Vierling LA, Weiss I, Muller J. Spinning a laser web: predicting spider distributions using LiDAR. Ecol Appl. 2011;21(2):577–88. 21563587 10.1890/09-2155.1

[22] Garibaldi LA, Muchhala N, Motzke I, Bravo-Monroy L, Olschewski R, Klein A-M. Services from Plant—Pollinator interactions in the Neotropics In: Rapidel B, DeClerck F, Le Coq J, Beer J, editors. Ecosystem services from agriculture and agroforestry: measurement and payment. London, UK: Earthscan; 2011 p. 119–39.

[23] Allen-Wardell G, Bernhardt P, Bitner R, Burquez A, Buchmann S, Cane J, et al The potential consequences of pollinator declines on the conservation of biodiversity and stability of food crop yields. Conserv Biol. 1998;12:8–17.

[24] Roulston TH, Goodell K. The role of resources and risks in regulating wild bee populations. Annu Rev Entomol. 2011;56:293–312. doi: 10.1146/annurev-ento-120709-144802 20822447 10.1146/annurev-ento-120709-144802

[25] Batary P, Baldi A, Kleijn D, Tscharntke T. Landscape-moderated biodiversity effects of agri-environmental management: a meta-analysis. Proceedings of Biological Science. 2011;278(1713):1894–902. 10.1098/rspb.2010.1923 PMC3097824 21106585

[26] Holzschuh A, Steffan-Dewenter I, Kleijn D, Tscharntke T. Diversity of flower-visiting bees in cereal fields: effects of farming system, landscape composition and regional context. J Appl Ecol. 2007;44(1):41–9.

[27] Boscolo D, Tokumoto PM, Ferreira PA, Ribeiro JW, DosSantos JS. Positive responses of flower visiting bees to landscape heterogeneity depend on functional connectivity levels. Perspectives in Ecology and Conservation. 2017;15(1):18–24.

[28] Frenzel M, Everaars J, Schweiger O. Bird communities in agricultural landscapes: What are the current drivers of temporal trends? Ecol Indic. 2016;65:113–21.

[29] Duelli P, Obrist MK, Schmatz DR. Biodiversity evaluation in agricultural landscapes: above-ground insects. Agric Ecosyst Environ. 1999;74:33–64.

[30] Papanikolaou AD, Kühn I, Frenzel M, Schweiger O. Semi-natural habitats mitigate the effects of temperature rise on wild bees. J Appl Ecol. 2017;54:527–36.

[31] Schweiger O, Maelfait JP, Van Wingerden W, Hendrickx F, Billeter R, Speelmans M, et al Quantifying the impact of environmental factors on arthropod communities in agricultural landscapes across organizational levels and spatial scales. J Appl Ecol. 2005;42(6):1129–39.

[32] Masek JG, Vermote EF, Saleous N, Wolfe R, Hall FG, Huemmrich F, et al LEDAPS Landsat calibration, reflectance, atmospheric correction preprocessing code. Oak Ridge, Tennessee, USA: ORNL DAAC; 2012.

[33] Tuanmu M-N, Jetz W. A global, remote sensing-based characterization of terrestrial habitat heterogeneity for biodiversity and ecosystem modelling. Glob Ecol Biogeogr. 2015;24:1329–39.

[34] McGarigal K, Marks BJ. FRAGSTATS: spatial pattern analysis program for quantifying landscape structure. General Technical Report PNW-351. USDA Forest Service, 1995.

[35] Baker WL, Cai YM. The r.le programs for multiscale analysis of landscape structure using the Grass Geographical Information-System. Landscape Ecol. 1992;7(4):291–302.

[36] Hall-Beyer M. The GLCM Tutorial Home Page Calgary, Canada: University of Calgary; 2007 Available from: http://www.fp.ucalgary.ca/mhallbey/the_glcm.htm.

[37] Beyer HL. Geospatial Modelling Environment (Version 0.7.3.0). 2012.

[38] Blaszczynski JS. Landform characterization with geographic information systems. Photogramm Eng Remote Sensing. 1997;63(2):183–91.

[39] Riley SJ, D DS, Elliot R. A terrain ruggedness index that quantifies topographic heterogeneity. Intermt J Sci. 1999;5:1–4.

[40] Evans JS, Oakleaf J, Cushman SA, Theobald D. An ArcGIS Toolbox for Surface Gradient and Geomorphometric Modeling, version 2.0–0 2014. Available from: http://evansmurphy.wix.com/evansspatial.

[41] Anys H, Bannari A, He DC, Morin D, editors. Texture analysis for the mapping of urban areas using airborne MEIS-II images. Proceedings of the First International Airborne Remote Sensing Conference and Exhibition 3; 1994.

[42] Hall-Beyer M. The GLCM tutorial home page. http://www.fp.ucalgary.ca/mhallbey/tutorial.htm [March 2016].

[43] Patel D, Stonham TJ. Texture image classification and segmentation using RANK-order clustering 11th IAPR International Conference on Pattern Recognition; The Hague, Netherlands: IEEE Computer Society Press; 1992 p. 92–5.

[44] Warner T. Kernel-based Texture in Remote Sensing Image Classification. Geography Compass. 2011;5(10):781–98.

[45] Rocchini D, Delucchi L, Ricotta C, Ghisla A, Castellani C, Zorer R, et al. Measuring spatial diversity in a free algorithmic environment. FOSS4G; Barcelona, Spain2010.

[46] RCoreTeam. R: A language and environment for statistical computing Vienna, Austria: R Foundation for Statistical Computing; 2016 Available from: https://www.R-project.org/.

[47] Murray TE, Fitzpatrick Ú, Byrne A, Fealy R, Brown MJF, Paxton RJ. Local-scale factors structure wild bee communities in protected areas. J Appl Ecol. 2012; 49(5):998–1008.

[48] Riedinger V, Mitesser O, Hovestadt T, Steffan-Dewenter I, Holzschuh A. Annual dynamics of wild bee densities attractiveness and productivity effects of oilseed rape. Ecology. 2015; 96(5):1351–60. 26236848 10.1890/14-1124.1

[49] Hsieh TC, Ma KH, Chao A. iNEXT: iNterpolation and EXTrapolation for species diversity. R package version 2.0.12 URL: http://chao.stat.nthu.edu.tw/blog/software-download/. 2016.

[50] Chao A, Gotelli NJ, Hsieh TC, Sander EL, Ma KH, Colwell RK, et al Rarefaction and extrapolation with Hill numbers a framework for sampling and estimation in species diversity studies. Ecol Monogr. 2014; 84(1):45–67.

[51] Shannon CE. A mathematical theory of communication. The Bell System Technical Journal. 1948;27:379–423 and 623–56.

[52] >Lau MK. DiversitySampler: Functions for re-sampling a community matrix to compute diversity indices at different sampling levels. R package version 2.1. 2015. Available from: http://cran.r-project.org/web/packages/DiversitySampler/. 2015.

[53] Kruskal WH, Wallis WA. Use of ranks in one-criterion variance analysis. J Am Stat Assoc. 1952;47:583–621.

[54] Fox J, Weisberg S, Adler D, Bates D, Baud-Bovy G, Ellison S>, et al. Car: Companion to applied regression. R package version 2.1–3. http://www.CRAN.R-project.org/package=car. x ed. Vienna, Austria: R project; 2016.

[55] Ripley R, Venables W, Bates D, Hornik K, Gebhardt A, Firth D. MASS: Support functions and datasets for venables and Ripley's MASS. R package version 7.3–45. https://cran.r-project.org/web/packages/MASS/. 2016.

[56] Barton K. MuMIn: Multi-model inference. R package version 1.16–0. http://CRAN.R-project.org/package=MuMIn. 2016.

[57] Bjornstad ON. Ncf: Spatial nonparametric covariance functions. R package version 1.1–7. https://cran.r-project.org/web/packages/ncf/index.html. 2016.

[58] Pinheiro J, Bates D, DebRoy S, Sarkar D, authors E, S. H, et al. Nlme: Linear and nonlinear mixed effects models. R package version 3.1–128. http://CRAN.R-project.org/package=nlme. 2016.

[59] Burnham KP, Anderson DR. Model selection and multimodel inference: a practical information-theoretic approach New York, NY: Springer US; 2002.

[60] Zuur AF, Ieno EN, Elphick CS. A protocol for data exploration to avoid common statistical problems. Methods Ecol Evol. 2010;1(1):3–14.

[61] Bjornstad O, Falck W. Nonparametric spatial covariance functions: Estimation and testing. Environ Ecol Stat. 2001;8:53–70.

[62] Johnson JB, Omland KS. Model selection in ecology and evolution. Trends Ecol Evol. 2004;19:101–8. doi: 10.1016/j.tree.2003.10.013 16701236 10.1016/j.tree.2003.10.013

[63] Wallis CIB, Brehm G, Donoso DA, Fiedler JH, Paulsch D, Süßenbach D, et al Remote sensing improves prediction of tropical montane speciesdiversity but performance differs among taxa. Ecol Indic. 2017 doi: 10.1016/j.ecolind.2017.01.022

[64] Wood EM, Pidgeon AM, Radeloff VC, Keuler NS. Image Texture Predicts Avian Density and Species Richness. PLOS One. 2013;8(5): e63211 doi: 10.1371/journal.pone.0063211 23675463 10.1371/journal.pone.0063211 PMC3651168

[65] Kuemmerle T, Hostert P, St-Louis V, Radeloff VC. Using image texture to map farmland field size: a case study in Eastern Europe. J Land Use Sci. 2009;4(1–2):85–107.

[66] Steckel J, Westphal C, Peters MK, Bellach M, Rothenwoehrer C, Erasmi S, et al Landscape composition and configuration differently affect trap-nesting bees, wasps and their antagonists. Biol Conserv. 2014;172:56–64.

[67] Dufour A, Gadallah F, Wagner HH, Guisan A, Buttler A. Plant species richness and environmental heterogeneity in a mountain landscape: effects of variability and spatial configuration. Ecography. 2006;29(4):573–84.

[68] Coblentz DD, Riitters KH. Topographic controls on the regional-scale biodiversity of the south-western USA. J Biogeogr. 2004;31(7):1125–38.

[69] Arellano G, Umaña MN, Macía MJ, Loza MI, Fuentes A, Cala V, et al The role of niche overlap, environmental heterogeneity, landscape roughness and productivity in shaping species abundance distributions along the Amazon-Andes gradient. Glob Ecol Biogeogr. 2017;26(2):191–202.

[70] Duro DC, Coops NC, Wilder MA, Han T. Development of a large area biodiversity monitoring system driven by remote sensing. Prog Phys Geogr. 2007;31(3):1–26.

[71] Carmo FF, Campos IC, Jacobi CM. Effects of fine-scale surface heterogeneity on rock outcrop plant community structure. J Veg Sci. 2016;27(1):50–9.

[72] Luoto M, Virkkala R, Heikkinen RK, Rainio K. Predicting bird species richness using remote sensing in boreal agricultural-forest mosaics. Ecol Appl. 2004;14(6):1946–62.

[73] Marini L, Bona E, Kunin WE, Gaston KJ. Exploring anthropogenic and natural processes shaping fern species richness along elevational gradients. J Biogeogr. 2011;38(1):78–88.

[74] Wrbka T, Erb KH, Schulz NB, Peterseil J, Hahn C, Haberl H. Linking pattern and process in cultural landscapes. An empirical study based on spatially explicit indicators. Land Use Policy. 2004;21(3):289–306.

[75] Holzschuh A, Dormann CF, Tscharntke T, Staffan-Dewenter I. Mass-flowering crops enhance wild bee abundance. Oecologia. 2013;172:477–84. doi: 10.1007/s00442-012-2515-5 23114428 10.1007/s00442-012-2515-5 PMC3655217

[76] Torne-Noguera A, Rodrigo A, Arnan X, Osorio S, Barril-Graells H, da Rocha-Filho LC, et al Determinants of spatial distribution in a bee community: nesting resources, flower resources, and body size. PLOS One. 2014;9(5):e97255 doi: 10.1371/journal.pone.0097255 24824445 10.1371/journal.pone.0097255 PMC4019551

[77] Breitbach N, Tillmann S, Schleuning M, Grunewald C, Laube I, Steffan-Dewenter I, et al Influence of habitat complexity and landscape configuration on pollination and seed-dispersal interactions of wild cherry trees. Oecologia. 2012;168(2):425–37. doi: 10.1007/s00442-011-2090-1 21818655 10.1007/s00442-011-2090-1

[78] Gallardo-Cruz JA, Meave JA, Gonzalez EJ, Lebrija-Trejos EE, Romero-Romero MA, Perez-Garcia EA, et al Predicting Tropical Dry Forest Successional Attributes from Space: Is the Key Hidden in Image Texture? PLoS One. 2012;7(2). doi: 10.1371/journal.pone.0030506 22363443 10.1371/journal.pone.0030506 PMC3282724

[79] Chapman RN, Engle DM, Masters RE, Leslie DM. Grassland vegetation and bird communities in the southern Great Plains of North America. Agr Ecosyst Environ. 2004;104(3):577–85.

[80] Jayapal R, Qureshi Q, Chellam R. Importance of forest structure versus floristics to composition of avian assemblages in tropical deciduous forests of Central Highlands, India. Forest Ecol Manag. 2009;257(11):2287–95.

[81] Lee PY, Rotenberry JT. Relationships between bird species and tree species assemblages in forested habitats of eastern North America. J Biogeogr. 2005;32(7):1139–50.

