# Supplementary material for: Modelling patterns of pollinator species richness and diversity using satellite image texture
Source: PLoS One. 2017 Oct 3;12(10):e0185591. doi: 10.1371/journal.pone.0185591 (PMC5626433; doi:10.1371/journal.pone.0185591)

**S1 Figure. Workflow of the study.** We performed all analyses on (i) the whole community of all wild bees (dataset 'nohb') and the community split in two subsets; ii) bumble bees (data set 'bb'), and iii) solitary bees (data set 'sb').


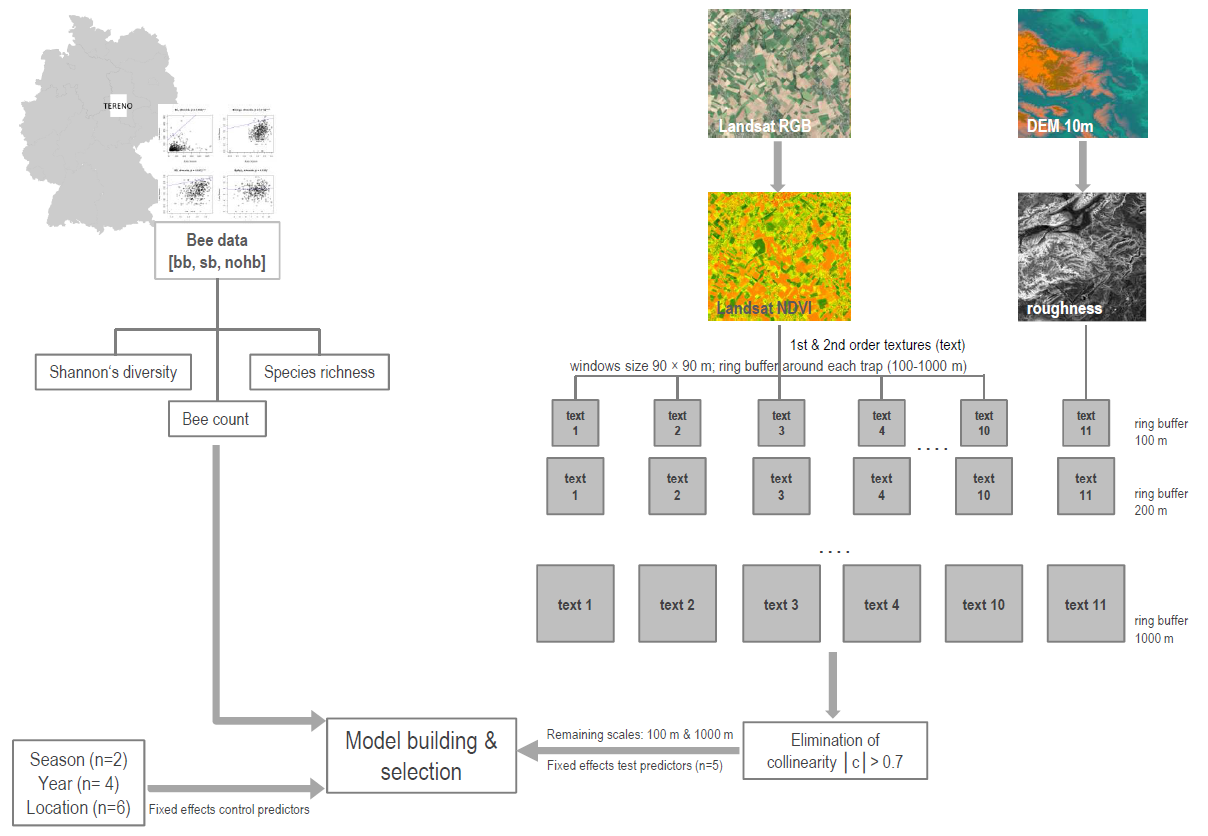

Supplement: S1 Fig — (DOCX) [file pone.0185591.s001.docx]
