# Supplementary material for: Modelling patterns of pollinator species richness and diversity using satellite image texture
Source: PLoS One. 2017 Oct 3;12(10):e0185591. doi: 10.1371/journal.pone.0185591 (PMC5626433; doi:10.1371/journal.pone.0185591)
Supplement: S2 Fig — (DOCX) [file pone.0185591.s002.docx]

**S2 Figure. Pearsons’s correlation coefficients for all pairs of distance classes per test variable.**

| 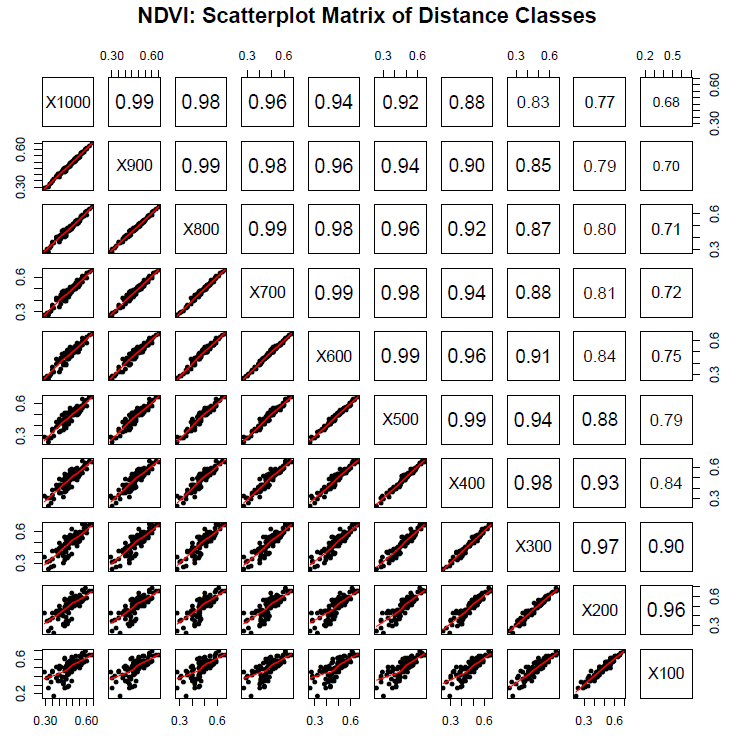 | 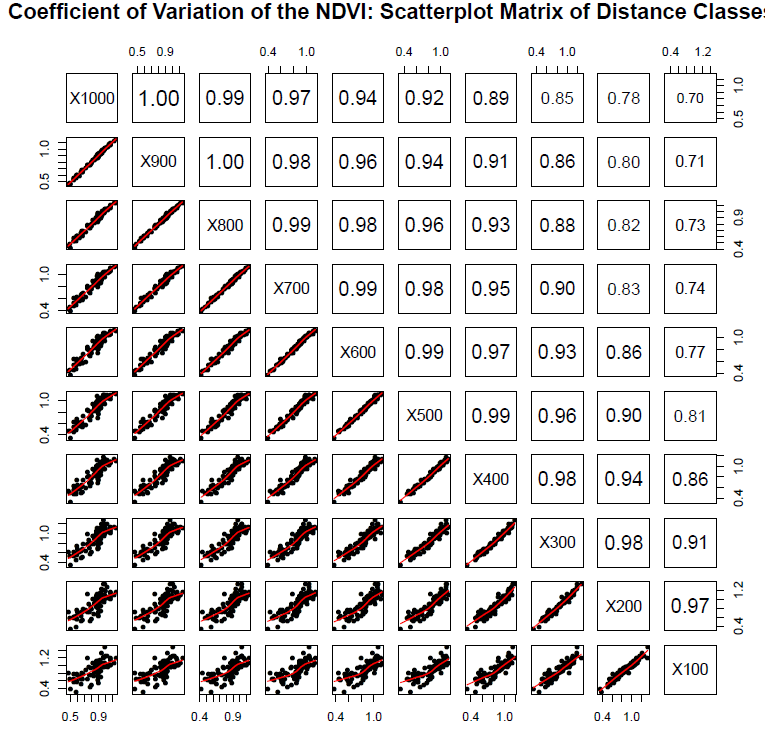 | 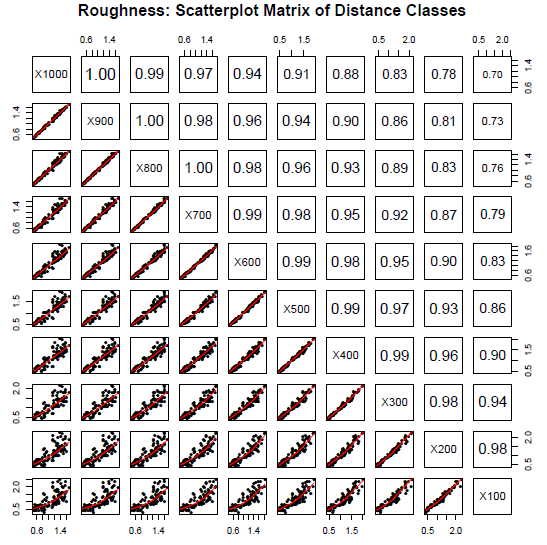 | 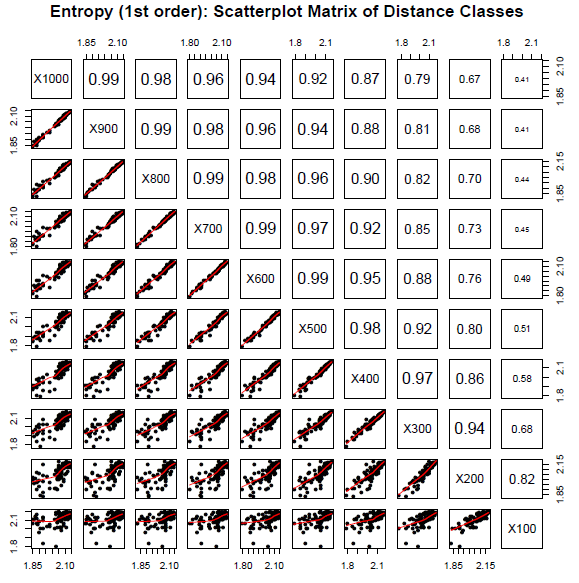 | 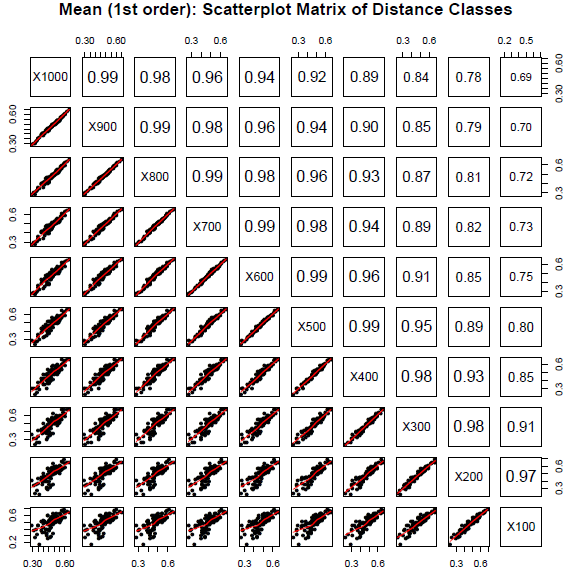 |
| --- | --- | --- | --- | --- |
| 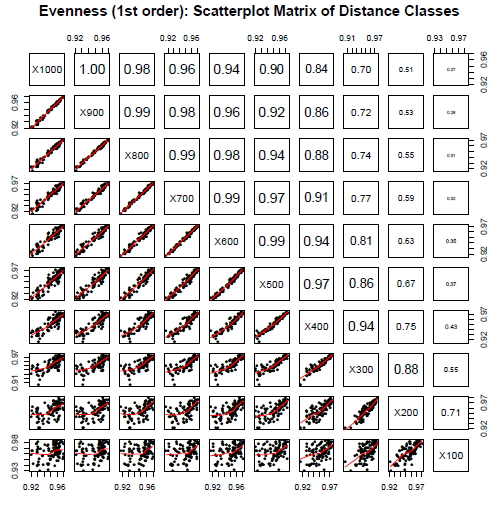 | 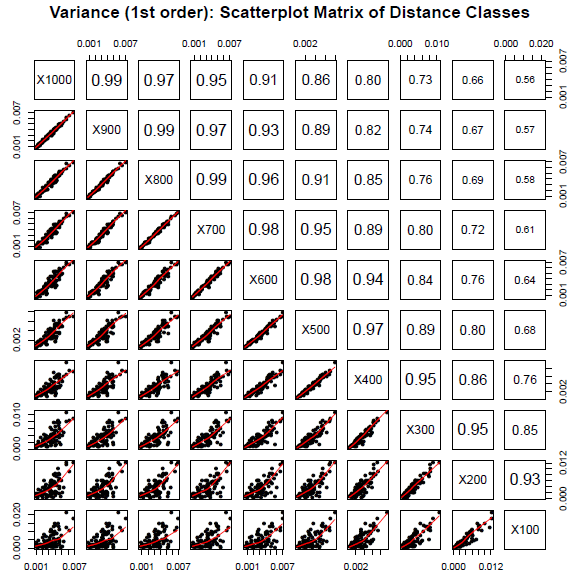 | 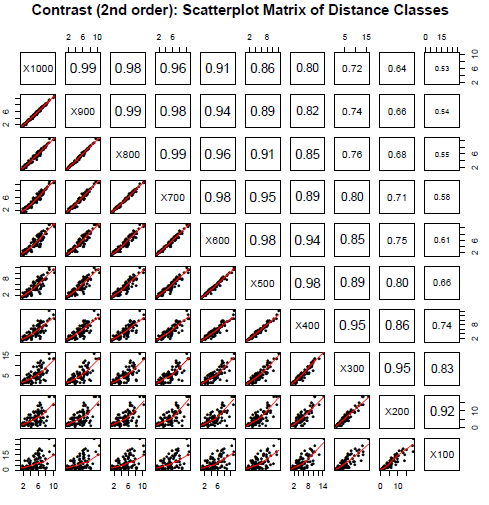 | 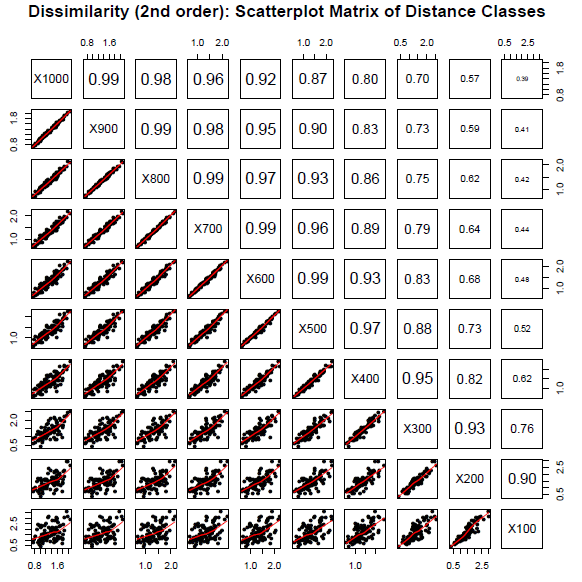 | 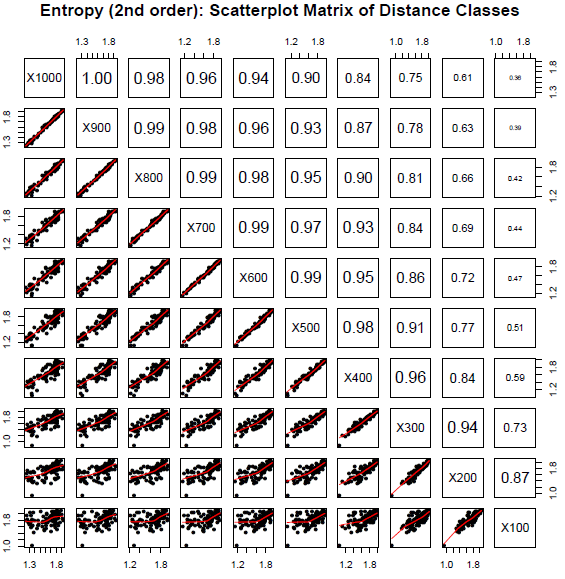 |
| 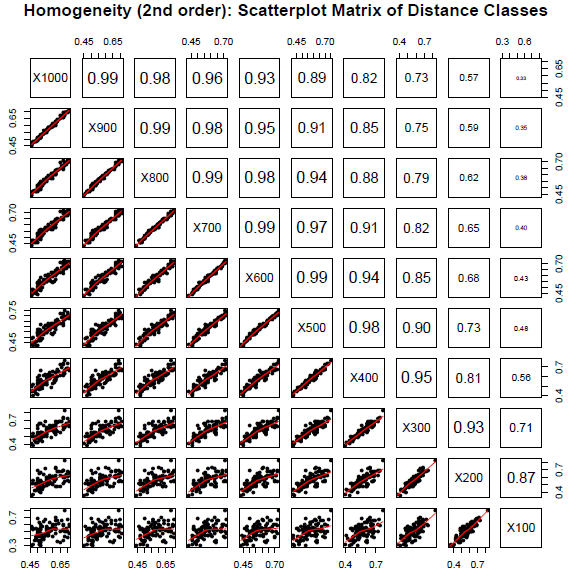 |  |  |  |  |
