# Supplementary material for: Modelling patterns of pollinator species richness and diversity using satellite image texture
Source: PLoS One. 2017 Oct 3;12(10):e0185591. doi: 10.1371/journal.pone.0185591 (PMC5626433; doi:10.1371/journal.pone.0185591)
Supplement: S3 Fig — Pearsons’s correlation coefficients between the test variables (a) and remaining variables after removing variables with coefficients │c│≥ 0.7 (b). (DOCX) [file pone.0185591.s003.docx]

**S3 Figure. Pearsons’s correlation coefficients between the test variables (a) and remaining variables after removing variables with coefficients │c│≥ 0.7 (b).** Variables that were excluded from subsequent analyses are crossed out. X1piel3MN=evenness (1^st^ order); X1ent3MN=entropy (1^st^ order); X1mw3MN=mean (1^st^ order); X1var3MN=variance (1^st^ order); X2con3MN=contrast (2^nd^ order); X2dis3MN= dissimilarity (2^nd^ order); X2ent3MN=entropy (2^nd^ order); X2hom3MN=homogeneity 2^nd^ order); NDVIMN=mean of NDVI; NDVI_cv= coefficient of variance of the NDVI; roug3MN=roughness.

| **a)**  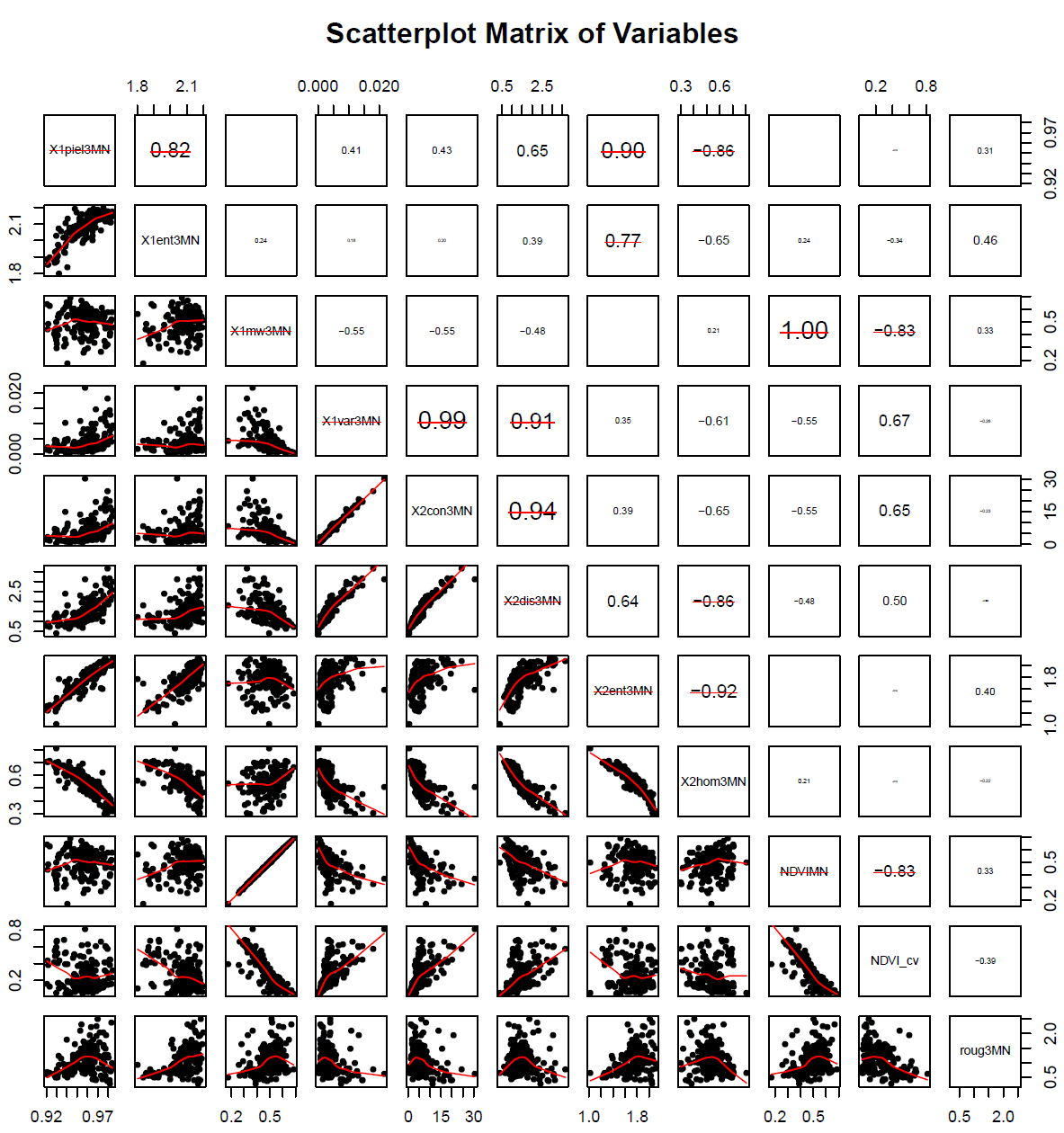 | **b)**  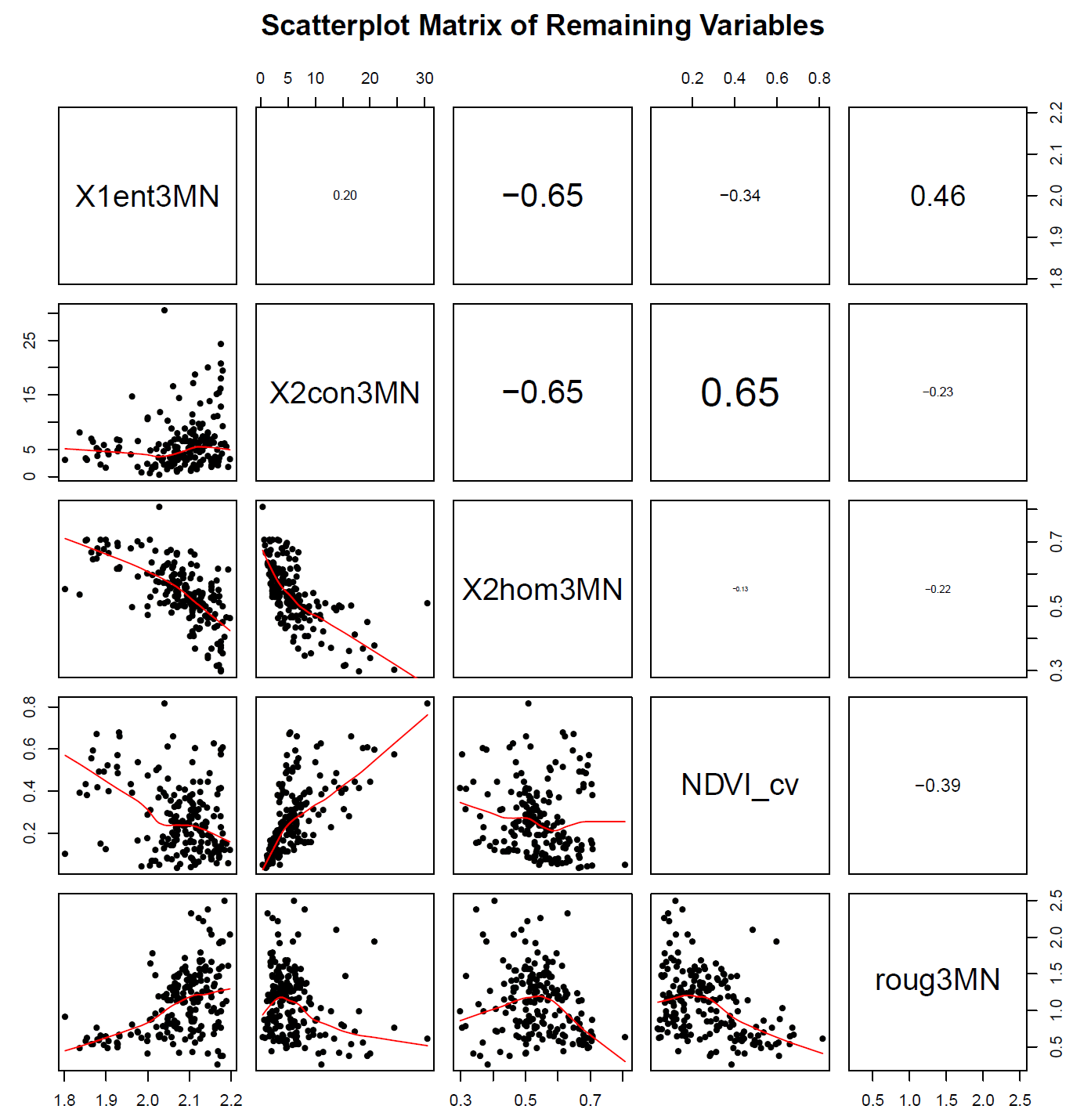 |
| --- | --- |
