# Supplementary material for: Modelling patterns of pollinator species richness and diversity using satellite image texture
Source: PLoS One. 2017 Oct 3;12(10):e0185591. doi: 10.1371/journal.pone.0185591 (PMC5626433; doi:10.1371/journal.pone.0185591)

**S4 Figure. Pearsons’s correlation coefficients for all pairs of remaining test variable and distance class (100 and 1000 m).** ent1_3MN_100(0)=entropy (1^st^ order), 100(0) m; con2_3MN_100(0)=contrast (2^nd^ order), 100(0) m; hom2_3MN_100(0)=homogeneity (2^nd^ order), 100(0) m; NDVI_cv_100(0)=coefficient of variance of the NDVI, 100(0) m; roug3MN_100(0)=roughness, 100(0) m.


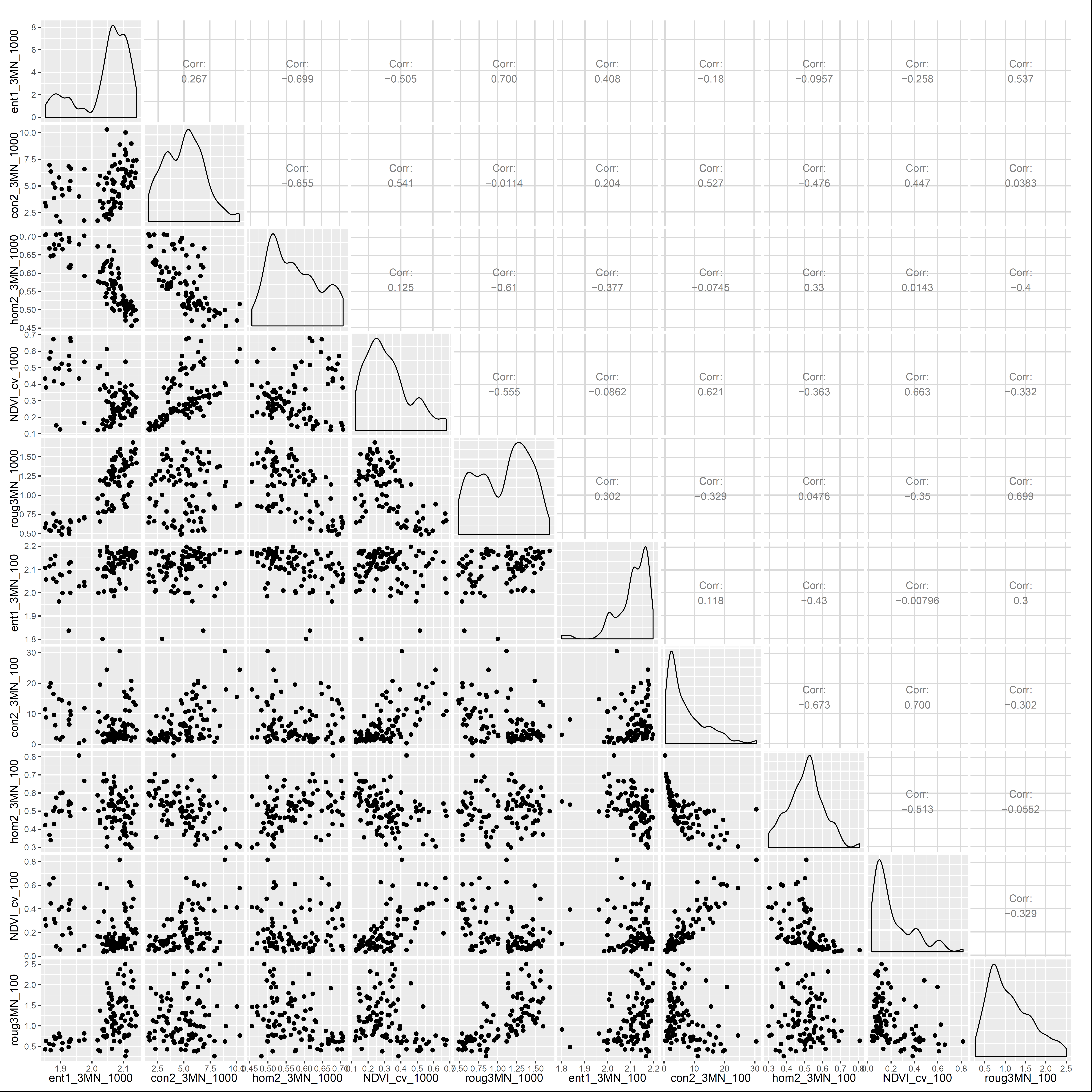

Supplement: S4 Fig — (DOCX) [file pone.0185591.s004.docx]
