# Supplementary material for: Modelling patterns of pollinator species richness and diversity using satellite image texture
Source: PLoS One. 2017 Oct 3;12(10):e0185591. doi: 10.1371/journal.pone.0185591 (PMC5626433; doi:10.1371/journal.pone.0185591)
Supplement: S5 Fig — (DOCX) [file pone.0185591.s005.docx]

**S5 Figure. Differences between trapping seasons (2010-23013) within the biodiversity variables per data set (df)**. Bee count, log-transformed bee count, Shannon’s diversity and species richness (corrected) are plotted for the bumble bees (bb), solitary bees (sb) and all wild bees (nohb); in each plot the minimum, 1^st^ quartil, median, 3^rd^ quartil and maximum are given. Differences between the seasons were highly significant according to Kruskal-Wallis tests (p < 0.01).

| 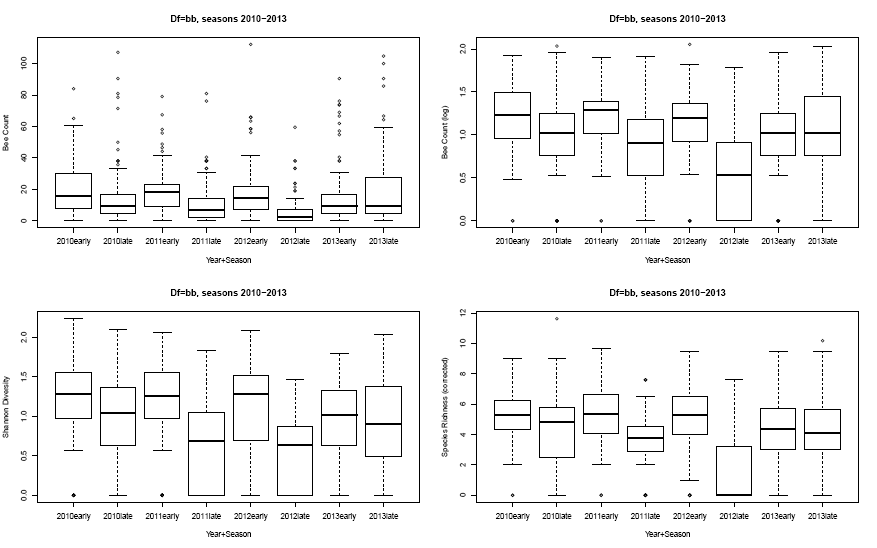 |
| --- |
| 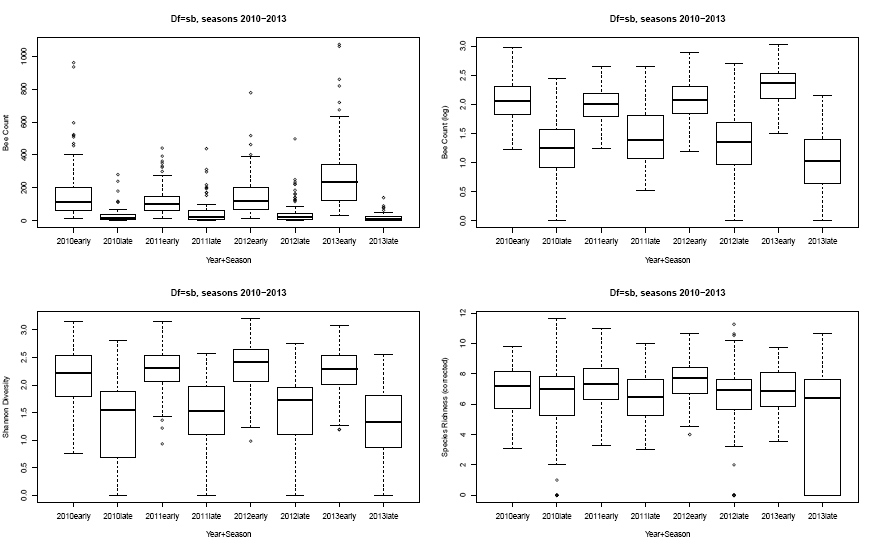 |
| 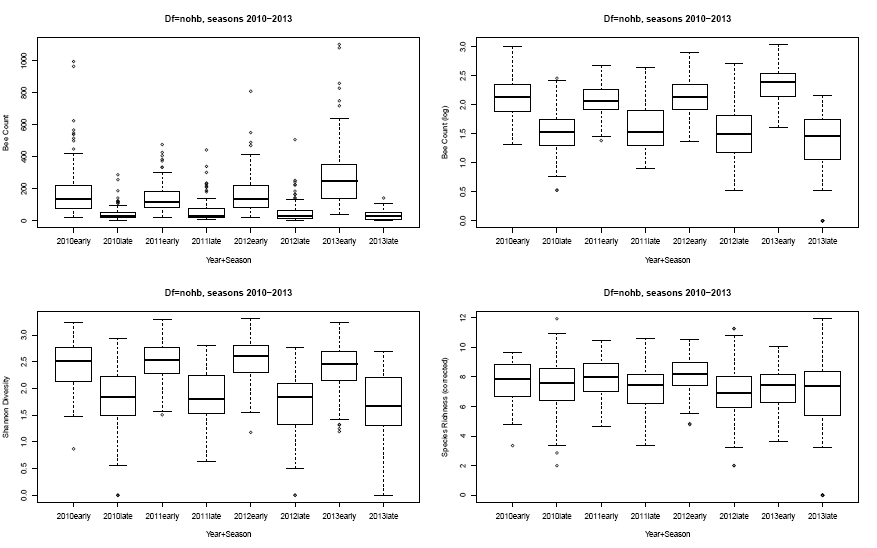 |
