# Supplementary material for: Modelling patterns of pollinator species richness and diversity using satellite image texture
Source: PLoS One. 2017 Oct 3;12(10):e0185591. doi: 10.1371/journal.pone.0185591 (PMC5626433; doi:10.1371/journal.pone.0185591)
Supplement: S6 Fig — (DOCX) [file pone.0185591.s006.docx]

**S6 Figure. Differences between years (upper two rows) and locations (lower two rows) within the biodiversity variables per data set (df).** Bee count, log-transformed bee count, Shannon diversity and species richness (corrected) are plotted for the bumble bees (bb), solitary bees (sb) and all wild bees (nohb); in each plot the minimum, 1^st^ quartil, median, 3^rd^ quartil and maximum are given. Biodiversity was highly significantly different between locations (p< 0.0001) but less significantly different or non-significant between years (p-values between 0.001 and 0.02) according to Kruskal-Wallis tests.

| 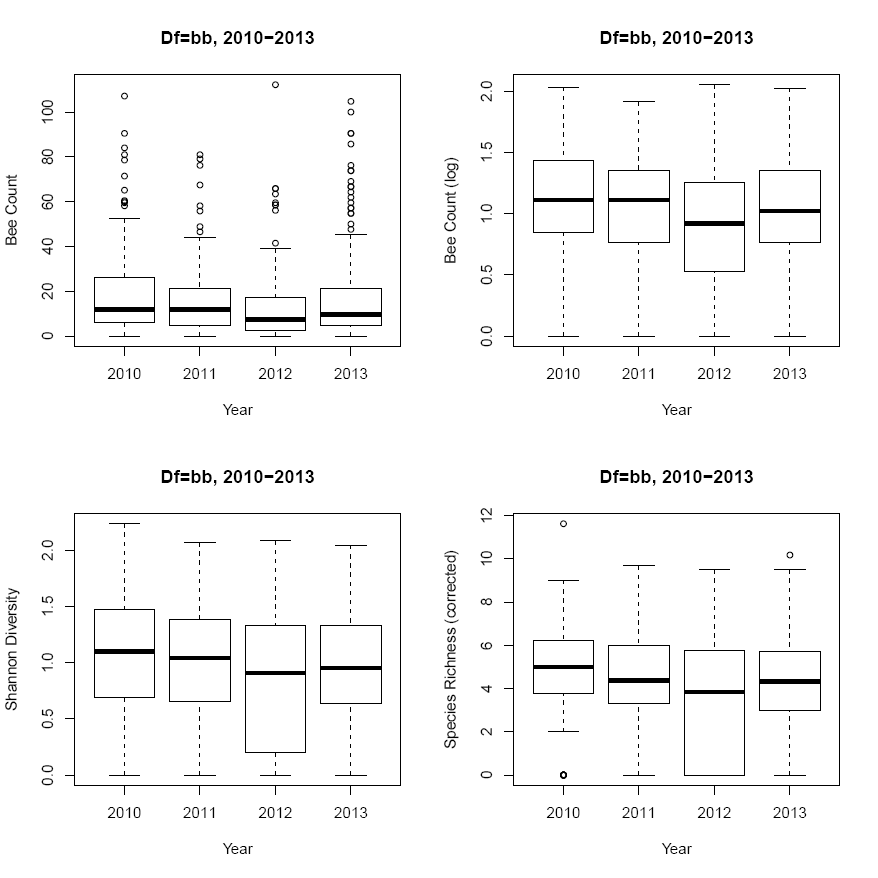 | 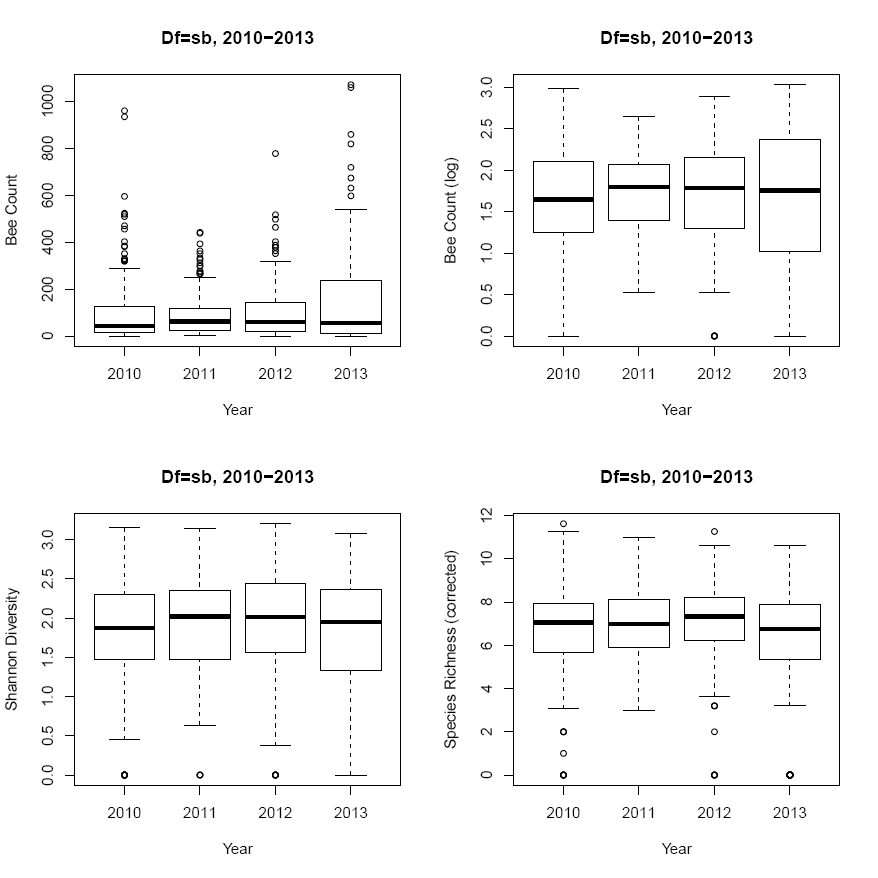 | 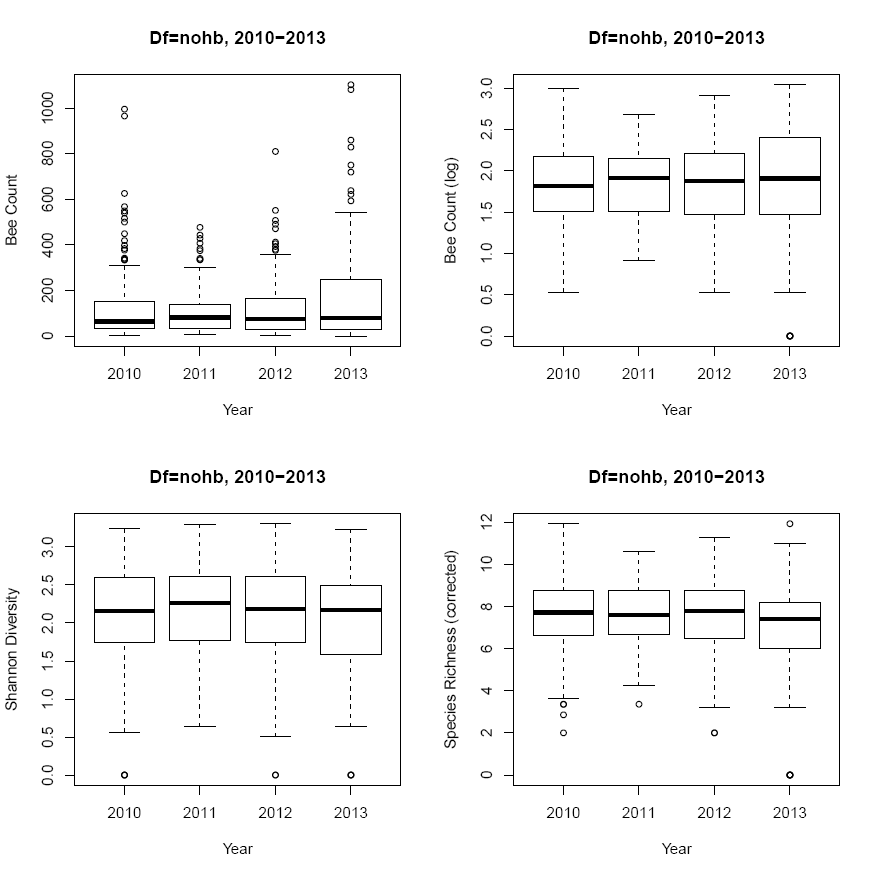 |
| --- | --- | --- |
| 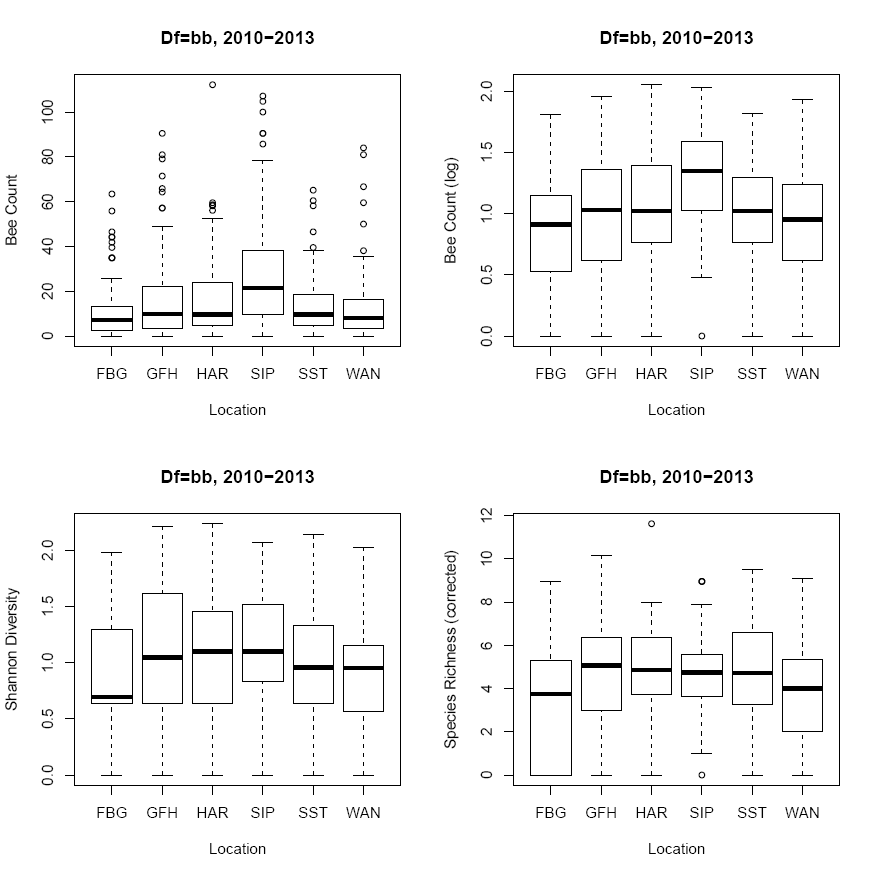 | 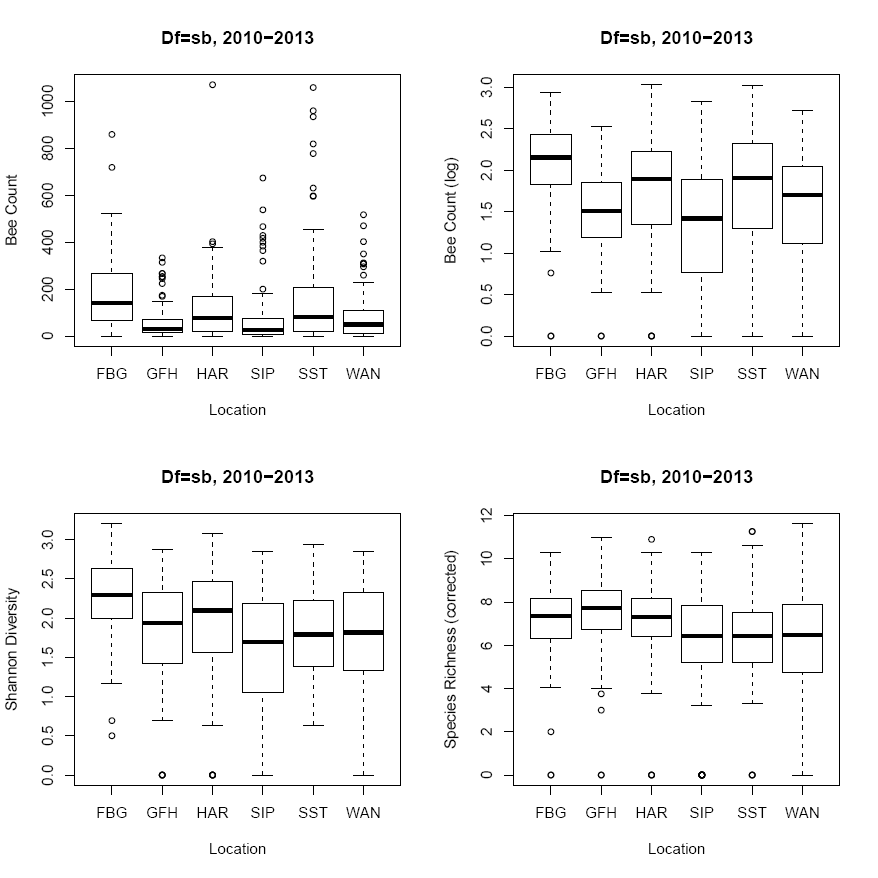 | 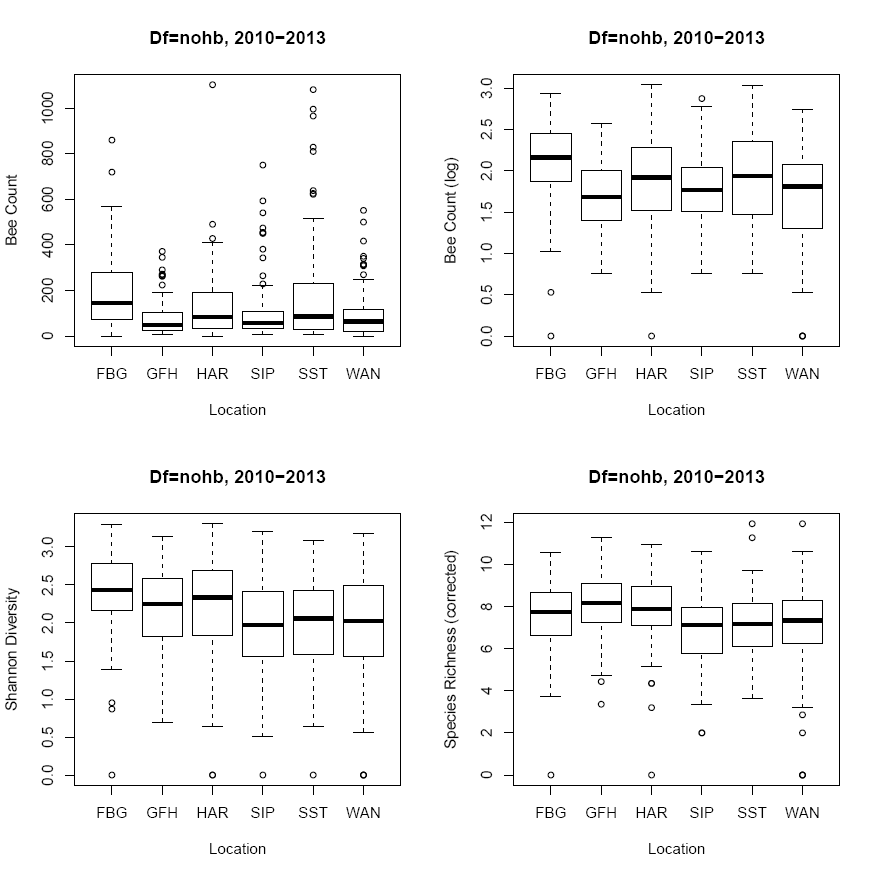 |
