# Supplementary material for: Modelling patterns of pollinator species richness and diversity using satellite image texture
Source: PLoS One. 2017 Oct 3;12(10):e0185591. doi: 10.1371/journal.pone.0185591 (PMC5626433; doi:10.1371/journal.pone.0185591)
Supplement: S7 Fig — (DOCX) [file pone.0185591.s007.docx]

**S7 Figure. Pearson’s correlation of the early and late trapping season for the biodiversity variables per data frame (df)**. Stars represent the levels of significance: **, p‑value between 0.001 and 0.01; ***, p-value between 0.0001 and 0.001; ****, p-value < 0.0001. Bclog = log-transformed bee count; SD = Shanonnon diversity; SpR(c) = corrected species richness; bb=bumble bees, sb=solitary bees, nohb=all wild bees.

| 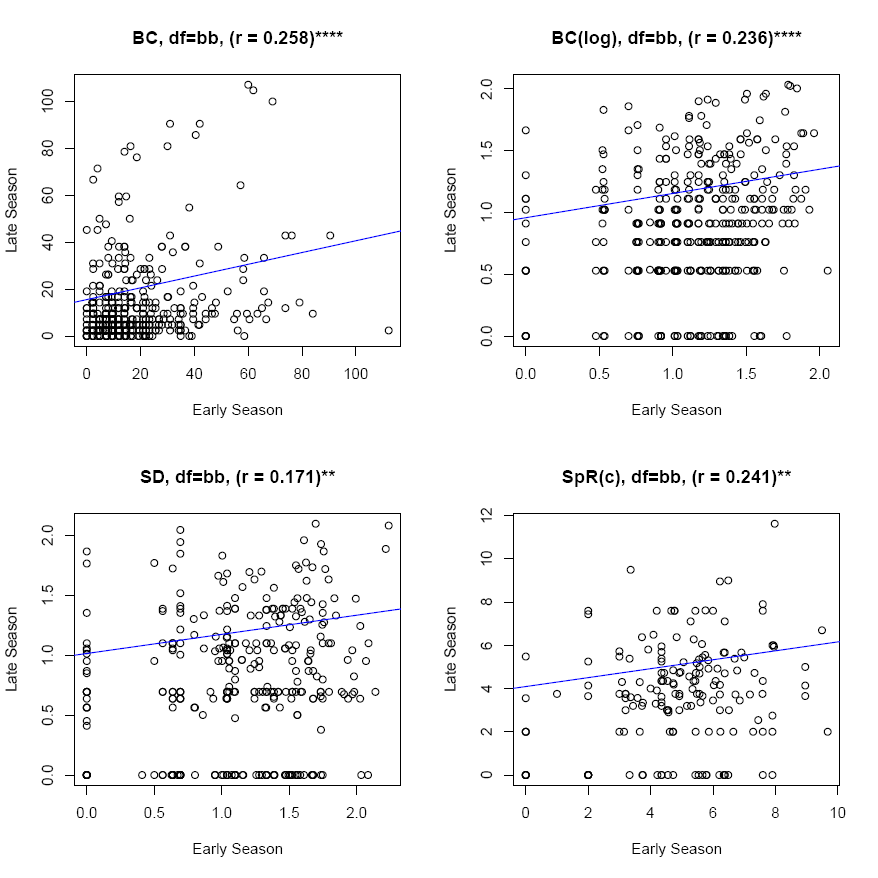 | 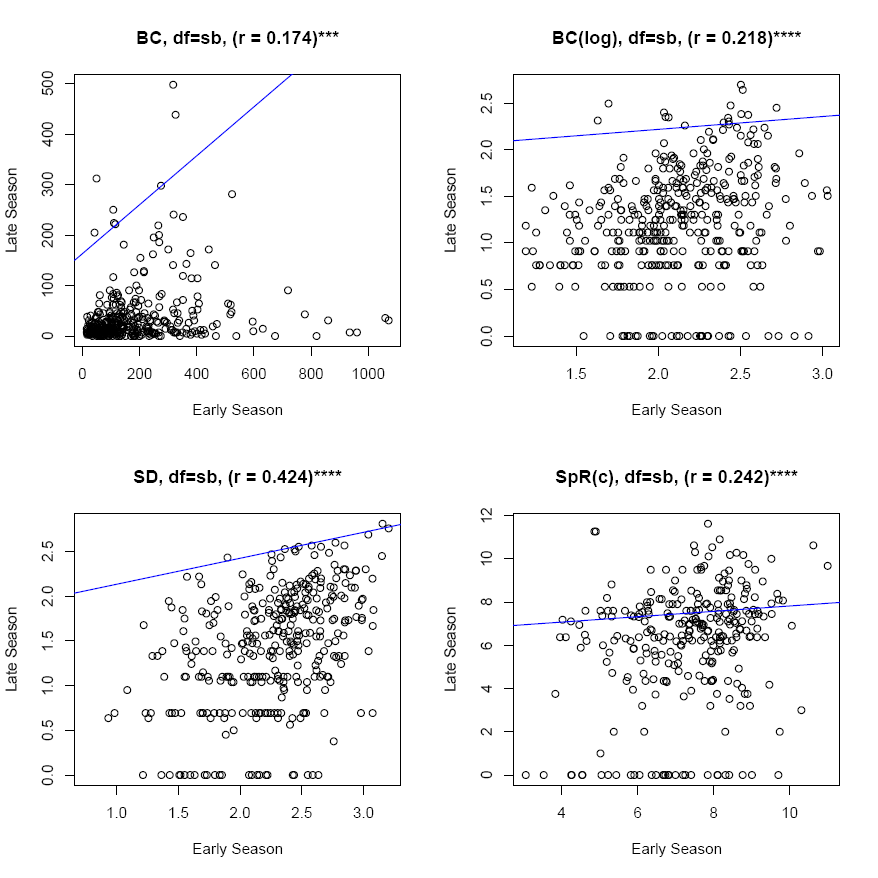 | 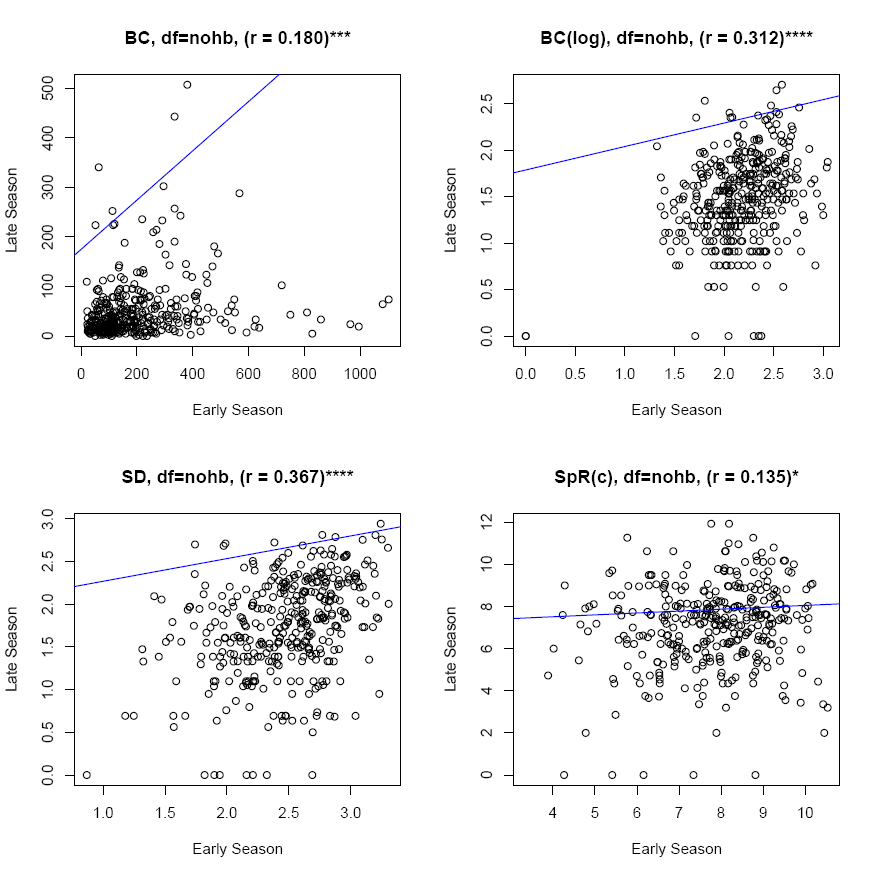 |
| --- | --- | --- |
