# Supplementary material for: Modelling patterns of pollinator species richness and diversity using satellite image texture
Source: PLoS One. 2017 Oct 3;12(10):e0185591. doi: 10.1371/journal.pone.0185591 (PMC5626433; doi:10.1371/journal.pone.0185591)
Supplement: S8 Fig — Spline correlograms for the global fitted models as per model selection for each response variable of (a) the bumble-bees data set (bb), (b) the solitary-bees data set (sb), and (c) the wild bee data set (nohb). (DOCX) [file pone.0185591.s008.docx]

**S8 Figure. Spline correlograms for the global fitted models as per model selection for each response variable of (a) the bumble-bees data set (bb), (b) the solitary-bees data set (sb), and (c) the wild bee data set (nohb).** No spatial structure is associated with the model residuals, confirming the data are spatially independent. Bclog = log-transformed bee count; SD = Shannon’s diversity; SpR(c) = corrected species richness; df = data set.

| **(a)**  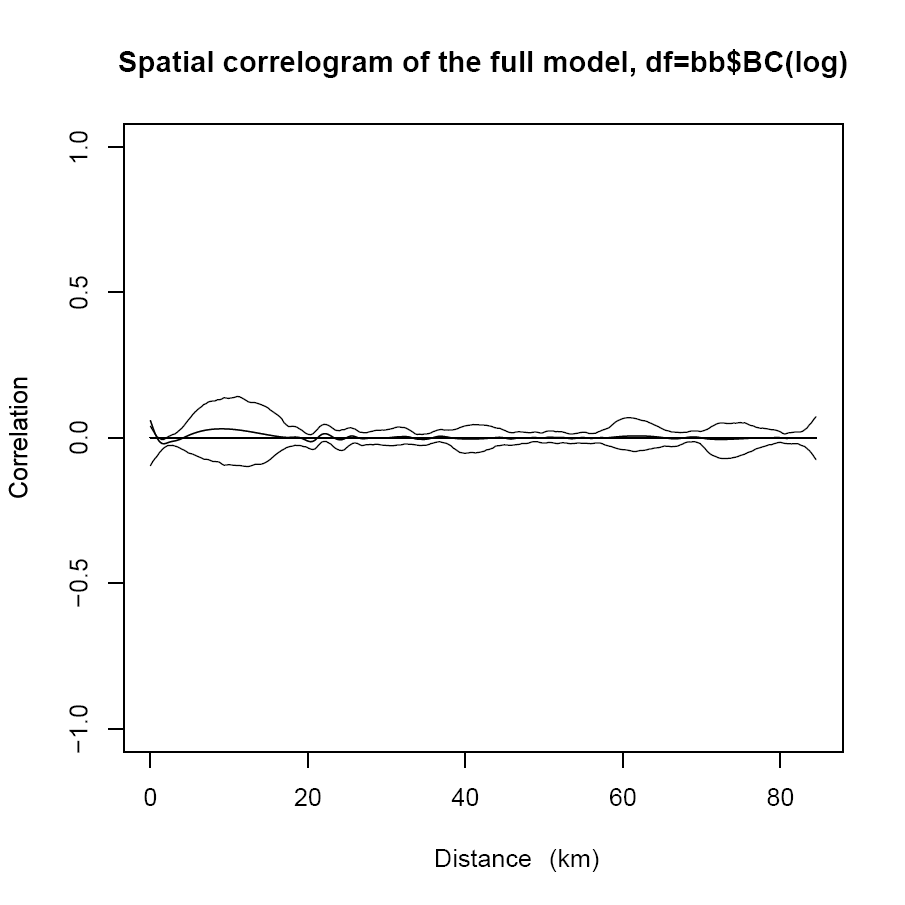 | 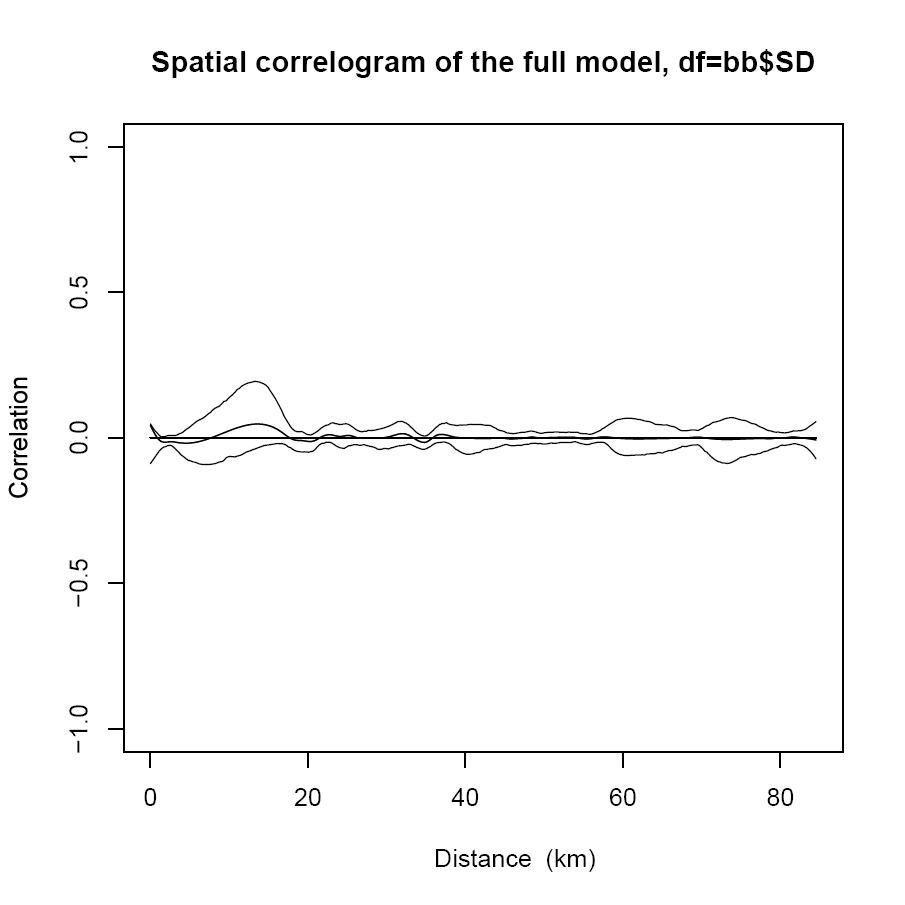 | 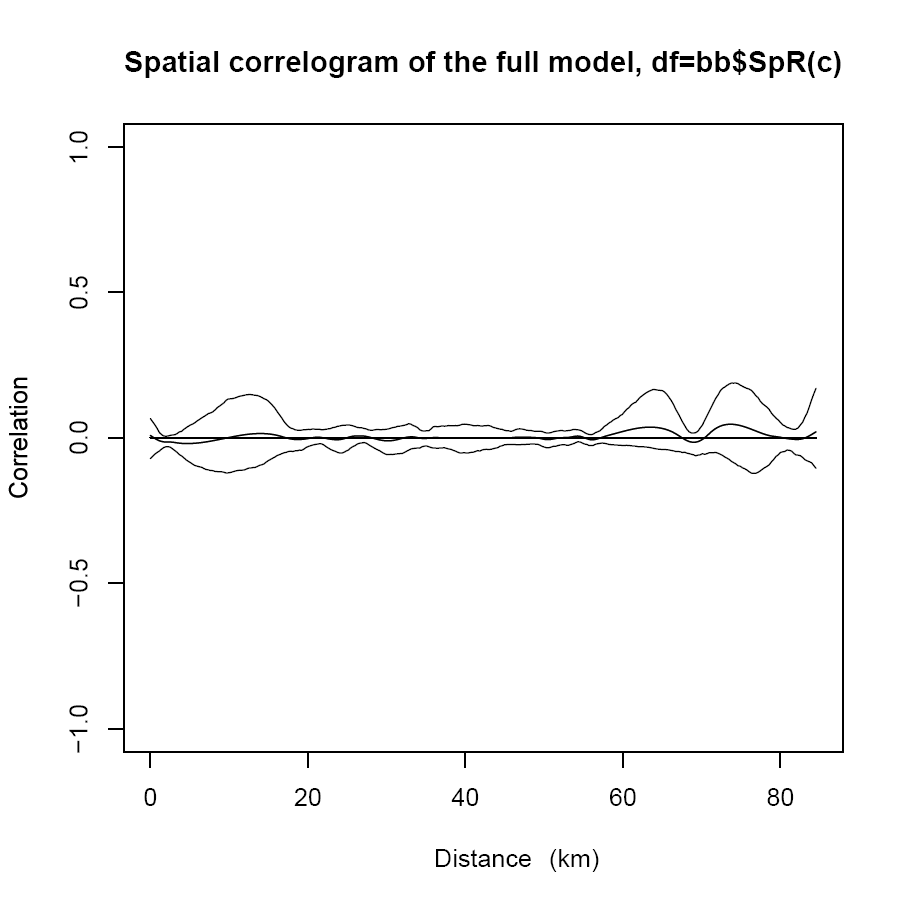 |
| --- | --- | --- |
| **(b)**  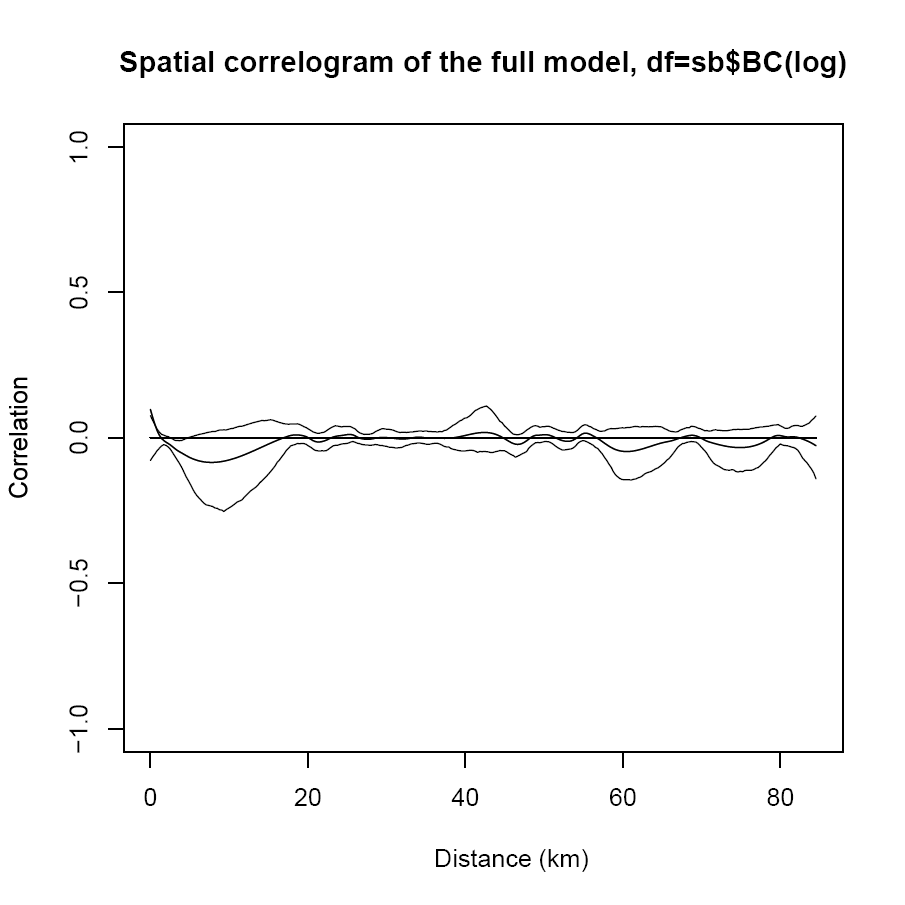 | 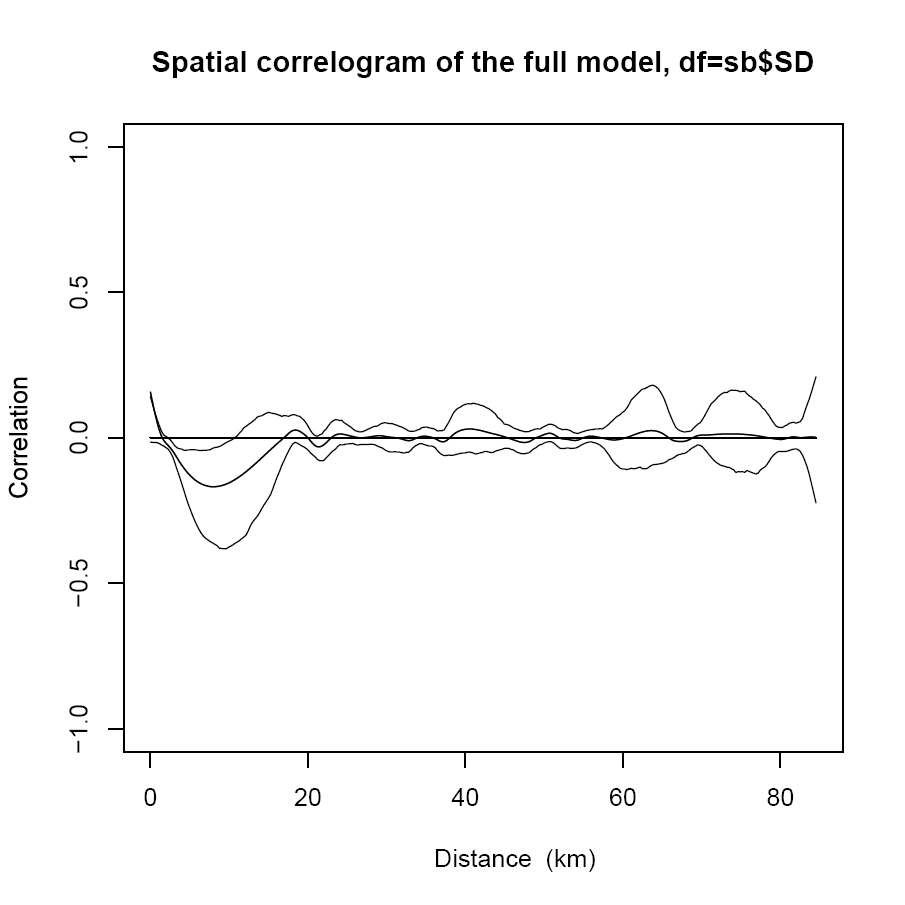 | 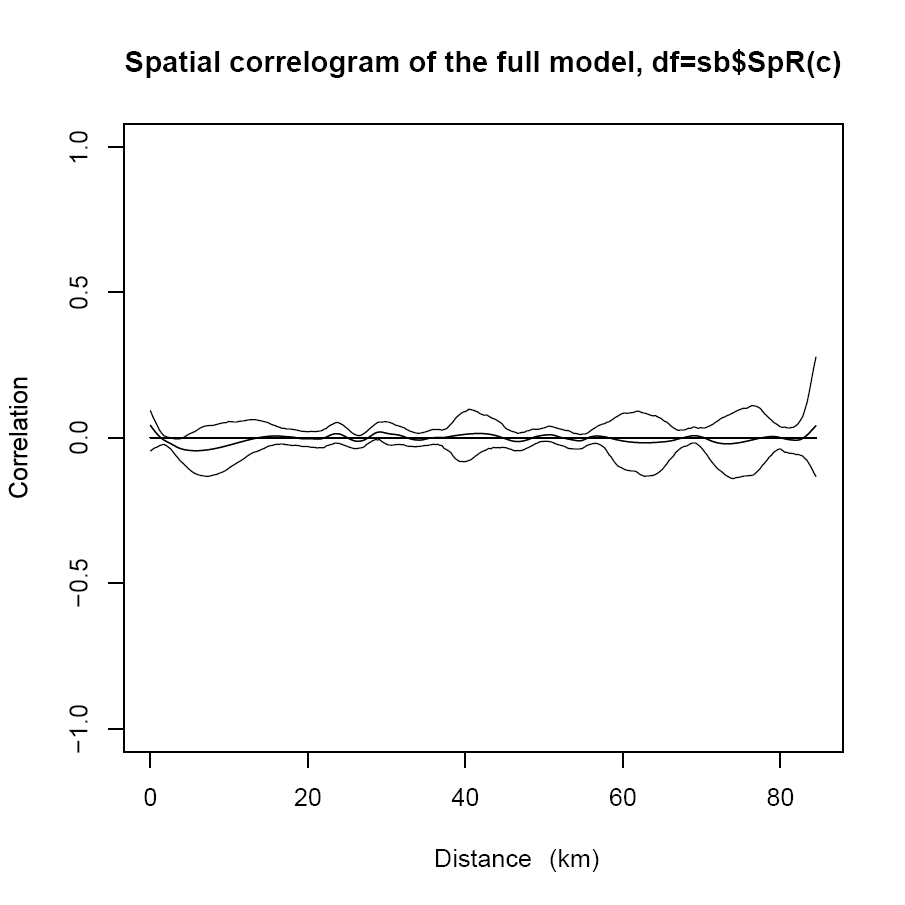 |
| **(c)**  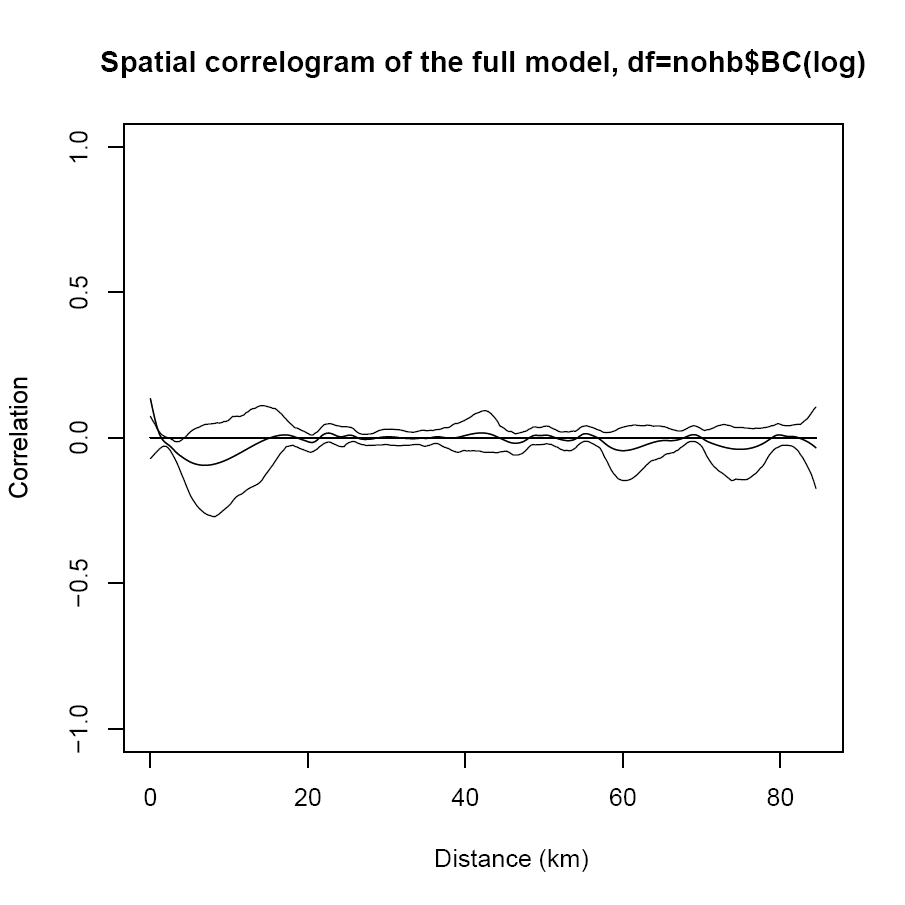 | 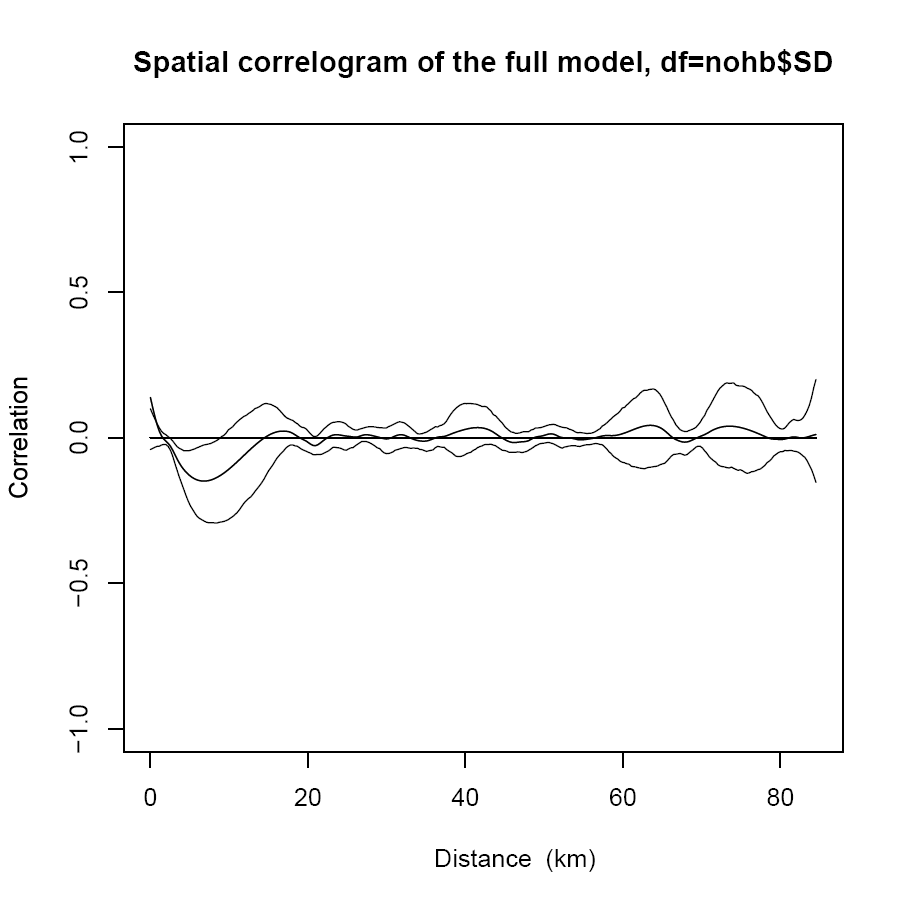 | 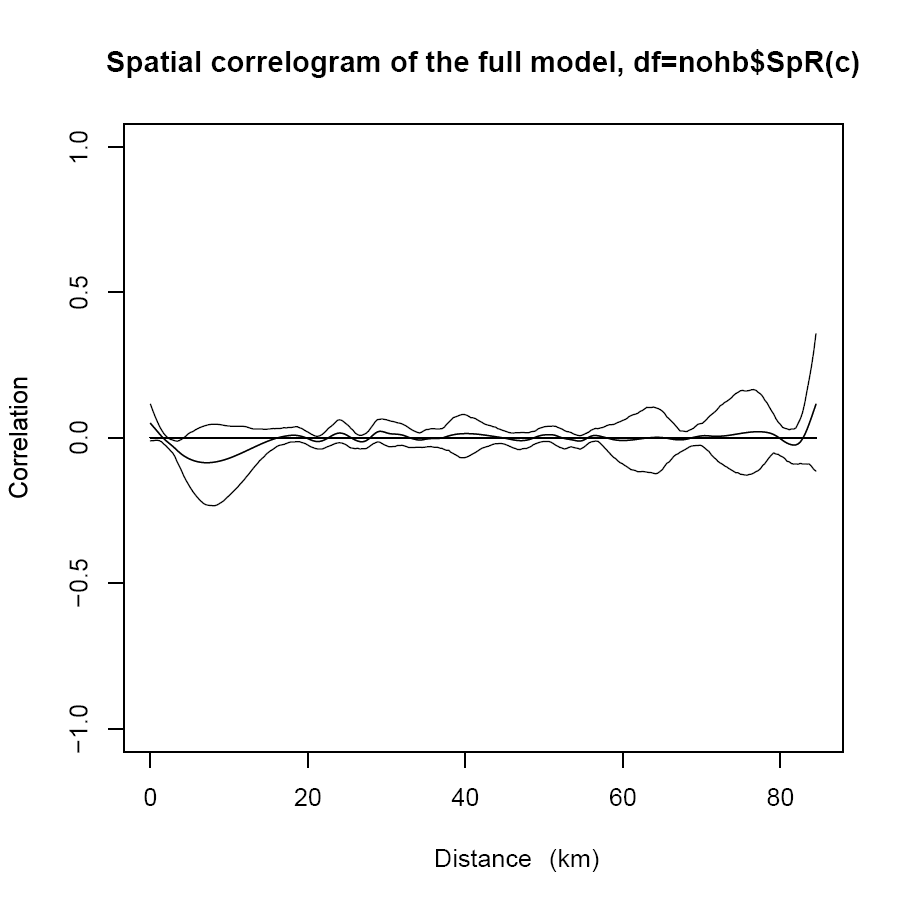 |
