# Supplementary material for: Modelling patterns of pollinator species richness and diversity using satellite image texture
Source: PLoS One. 2017 Oct 3;12(10):e0185591. doi: 10.1371/journal.pone.0185591 (PMC5626433; doi:10.1371/journal.pone.0185591)
Supplement: S1 Table — (DOCX) [file pone.0185591.s009.docx]

**S1 Table.** **Correlation between bee count (BC), Shannon’s diversity (SD) and species richness (SpR) variables.** The strength of the correlation was tested using Pearson’s method and Bonferroni-adjustment for multiple testing implemented in function corr.test from the R-package *psych*. Upper matrix: P-values; lower matrix: Pearson’s r. bb= bumble bees; nohb=all wild bees; sb=solitary bees.

| **bb** | *BC* | *SD* | *SpR* |  | **nohb** | *BC* | *SD* | *SpR* |  | **sb** | *BC* | *SD* | *SpR* |
| --- | --- | --- | --- | --- | --- | --- | --- | --- | --- | --- | --- | --- | --- |
| *BC* | – | <0.05 | <0.05 |  | *BC* | – | <0.05 | 0.130 |  | *BC* | – | <0.05 | 0.140 |
| *SD* | 0.605 | – | <0.05 |  | *SD* | 0.420 | – | <0.05 |  | *SD* | 0.057 | – | <0.05 |
| *SpR* | 0.357 | 0.862 | – |  | *SpR* | -0.056 | 0.713 | – |  | *SpR* | 0.444 | 0.716 | – |
