# Supplementary material for: Modelling patterns of pollinator species richness and diversity using satellite image texture
Source: PLoS One. 2017 Oct 3;12(10):e0185591. doi: 10.1371/journal.pone.0185591 (PMC5626433; doi:10.1371/journal.pone.0185591)
Supplement: S2 Table — (DOCX) [file pone.0185591.s010.docx]

**S2 Table. Model comparison for predictors of biodiversity in bumble bees (bb), solitary bees (sb), and all wild bees (nohb).** 1=intercept; loc=location; sn=season; y=year; en1²=first order entropy within a 100 m radius; hm1²=second order homogeneity within a 100 m radius; NDVIcv1²^/^³=coefficient of variance of the NDVI within a 100 m/1000 m radius; rg1²=surface roughness within a 100 m radius.

| *bb:BC (log) model* | *Number of parameters* | *AIC* | *ΔAIC <2* | *AIC weight* | *log likelihood* |
| --- | --- | --- | --- | --- | --- |
| 1+loc+sn+y+en1²+hm1²+rg1² | 14 | 850.5 | 0.00 | 0.195 | -410.955 |
| 1+loc+sn+y+cn1³+en1²+hm1²+rg1² | 15 | 850.8 | 0.29 | 0.169 | -410.060 |
| 1+loc+sn+y+cn1³+en1²+hm1²+NDVIcv1³+rg1² | 16 | 851.3 | 0.82 | 0.129 | -409.283 |
| 1+loc+sn+y+en1²+hm1²+NDVIcv1²+rg1² | 15 | 852.1 | 1.58 | 0.088 | -410.704 |
| 1+loc+sn+y+cn1³+en1³+hm1²+NDVIcv1²+NDVIcv1³+rg1² | 17 | 852.2 | 1.71 | 0.083 | -408.682 |
| 1+loc+sn+y+en1³+hm1²+NDVIcv1³+rg1² | 15 | 852.3 | 1.83 | 0.078 | -410.828 |
| *bb: SD model* | *Number of parameters* | *AIC* | *ΔAIC <2* | *AIC weight* | *log likelihood* |
| 1+loc+sn+ y+en1²+rg1² | 13 | 1086.7 | 0.00 | 0.138 | -530.080 |
| 1+loc+sn+y+cn1³+en1²+NDVIcv1³+rg1² | 15 | 1086.8 | 0.13 | 0.129 | -528.062 |
| 1+loc+sn+y+cn1³+en1²+rg1² | 14 | 1087.3 | 0.61 | 0.102 | -529.346 |
| 1+loc+sn+y+en1²+hm1²+rg1² | 14 | 1087.8 | 1.11 | 0.080 | -529.592 |
| 1+loc+sn+y+cn1³+en1²+hm1²+NDVIcv1³+rg1² | 16 | 1087.9 | 1.25 | 0.074 | -527.572 |
| 1+loc+sn+y+en1²+NDVIcv1³+rg1² | 14 | 1088.2 | 1.52 | 0.065 | -529.799 |
| 1+loc+sn+y+en1²+hm1²+NDVIcv1³+rg1² | 15 | 1088.6 | 1.87 | 0.054 | -528.928 |
| 1+loc+sn+y+cn1³+en1²+NDVIcv1²+NDVIcv1³+rg1² | 16 | 1088.6 | 1.93 | 0.053 | -527.914 |
| *bb: SpR model* | *Number of parameters* | *AIC* | *ΔAIC <2* | *AIC weight* | *log likelihood* |
| 1+loc+sn+y+rg1² | 12 | 2205.6 | 0.00 | 0.154 | -1090.496 |
| 1+loc+sn+y+NDVIcv²+rg1² | 13 | 2207.0 | 1.38 | 0.077 | -1090.135 |
| 1+loc+sn+y+en1²+rg1² | 13 | 2207.1 | 1.47 | 0.074 | -1090.177 |
| 1+loc+sn+y+NDVIcv³+rg1² | 13 | 2207.5 | 1.83 | 0.062 | -1090.355 |
| 1+loc+sn+y+hm1²+rg1² | 13 | 2207.5 | 1.89 | 0.060 | -1090.388 |
| 1+loc+sn+y+NDVIcv²+NDVIcv³+rg1² | 14 | 2207.6 | 1.98 | 0.057 | -1089.375 |
| *sb:BC (log) model* | *Number of parameters* | *AIC* | *ΔAIC <2* | *AIC weight* | *log likelihood* |
| 1+loc+sn+y+en1²+NDVIcv1²+rg1² | 14 | 858.4 | 0.00 | 0.081 | -414.916 |
| 1+loc+sn+y+en1²+NDVIcv1² | 13 | 858.4 | 0.20 | 0.074 | -416.055 |
| 1+loc+sn+y+en1²+rg1² | 13 | 858.4 | 0.28 | 0.071 | -416.092 |
| 1+loc+sn+y+en1²+hm1²+rg1² | 14 | 858.4 | 0.63 | 0.059 | -415.231 |
| 1+loc+sn+y+en1³+hm1²+NDVIcv1²+rg1² | 15 | 858.4 | 1.73 | 0.034 | -414.742 |
| 1+loc+sn+y+en1³+NDVIcv1²+NDVIcv1³+rg1² | 15 | 858.4 | 1.89 | 0.032 | -414.822 |
| 1+loc+sn+y+en1³+NDVIcv1²+NDVIcv1³ | 14 | 858.4 | 1.93 | 0.031 | -415.882 |
| 1+loc+sn+y+en1³ | 12 | 858.4 | 1.95 | 0.031 | -417.966 |
| *sb: SD model* | *Number of parameters* | *AIC* | *ΔAIC <2* | *AIC weight* | *log likelihood* |
| 1+loc+sn+y+en1²+hm1²+rg1² | 14 | 1050.7 | 0.00 | 0.151 | -511.031 |
| 1+loc+sn+y+en1²+NDVIcv1²+rg1² | 14 | 1050.9 | 0.28 | 0.131 | -511.171 |
| 1+loc+sn+y+en1²+rg1² | 13 | 1051.2 | 0.60 | 0.112 | -512.369 |
| 1+loc+sn+y+en1²+hm1²+NDVIcv1³+rg1² | 15 | 1052.1 | 1.44 | 0.073 | -510.709 |
| 1+loc+sn+y+cn1³+en1²+hm1²+rg1² | 15 | 1052.3 | 1.67 | 0.066 | -510.822 |
| 1+loc+sn+y+cn1³+en1²+NDVIcv1²+rg1² | 15 | 1052.6 | 1.91 | 0.058 | -510.943 |
| 1+loc+sn+y+en1²+NDVIcv1³+rg1² | 14 | 1052.6 | 1.93 | 0.058 | -511.994 |
| 1+loc+sn+y+en1²+hm1²+NDVIcv1³+rg1² | 15 | 1052.6 | 1.97 | 0.056 | -510.975 |
| *sb: SpR model* | *Number of parameters* | *AIC* | *ΔAIC <2* | *AIC weight* | *log likelihood* |
| 1+loc+sn+y+cn1³+en1²+NDVIcv1³+rg1² | 15 | 2821.9 | 0.00 | 0.129 | -1395.596 |
| 1+loc+sn+y+en1²+rg1² | 13 | 2822.6 | 0.72 | 0.090 | -1398.047 |
| 1+loc+sn+y+cn1³+en1²+hm1²+NDVIcv1³+rg1² | 16 | 2822.9 | 0.94 | 0.080 | -1395.015 |
| 1+loc+sn+y+en1²+NDVIcv³+rg1² | 14 | 2822.9 | 0.98 | 0.079 | -1397.135 |
| 1+loc+sn+y+en1²+hm1²+rg1² | 14 | 2823.4 | 1.49 | 0.061 | -1397.386 |
| 1+loc+sn+y+cn1³+en1²+hm1²+rg1² | 15 | 2823.8 | 1.92 | 0.049 | -1396.558 |

| *nohb:BC (log) model* | *Number of parameters* | *AIC* | *ΔAIC <2* | *AIC weight* | *log likelihood* |
| --- | --- | --- | --- | --- | --- |
| 1+loc+sn+y+en1²+ NDVIcv1²+rg1² | 11 | 623.7 | 0.00 | 0.167 | -300.690 |
| 1+loc+sn+y+en1²+hm1²+NDVIcv1²+rg1² | 12 | 625.0 | 1.26 | 0.089 | -300.287 |
| 1+loc+sn+y+en1²+hm1²+rg1² | 11 | 625.3 | 1.52 | 0.078 | -301.450 |
| 1+loc+sn+y+en1³+NDVIcv1²+NDVIcv1³+rg1² | 12 | 625.5 | 1.75 | 0.070 | -300.531 |
| 1+loc+sn+y+cn1³+en1³+NDVIcv1²+rg1² | 12 | 625.6 | 1.86 | 0.066 | -300.588 |
| *nohb: SD model* | *Number of parameters* | *AIC* | *ΔAIC <2* | *AIC weight* | *log likelihood* |
| 1+loc+sn+y+en1²+hm1²+rg1² | 14 | 987.3 | 0.00 | 0.206 | -479.362 |
| 1+loc+sn+y+cn1³+en1²+hm1²+rg1² | 15 | 988.2 | 0.93 | 0.129 | -478.788 |
| 1+loc+sn+y+en1²+rg1² | 13 | 988.9 | 1.64 | 0.091 | -481.219 |
| *nohb: SpR model* | *Number of parameters* | *AIC* | *ΔAIC <2* | *AIC weight* | *log likelihood* |
| 1+loc+sn+y+en1²+rg1² | 13 | 2740.3 | 0.00 | 0.204 | -1356.896 |
| 1+loc+sn+y+en1²+NDVIcv³+rg1² | 14 | 2741.5 | 1.15 | 0.115 | -1356.432 |
| 1+loc+sn+y+en1²+hm1²+rg1² | 14 | 2741.7 | 1.34 | 0.104 | -1356.528 |
| 1+loc+sn+y+en1²+NDVIcv²+rg1² | 14 | 2742.0 | 1.72 | 0.087 | -1356.714 |
